# Supplementary figures and images for: Moving in on human motor cortex. Characterizing the relationship between body parts with non-rigid population response fields
Source: PLoS Comput Biol. 2022 Apr 4;18(4):e1009955. doi: 10.1371/journal.pcbi.1009955 (PMC9009778; doi:10.1371/journal.pcbi.1009955)

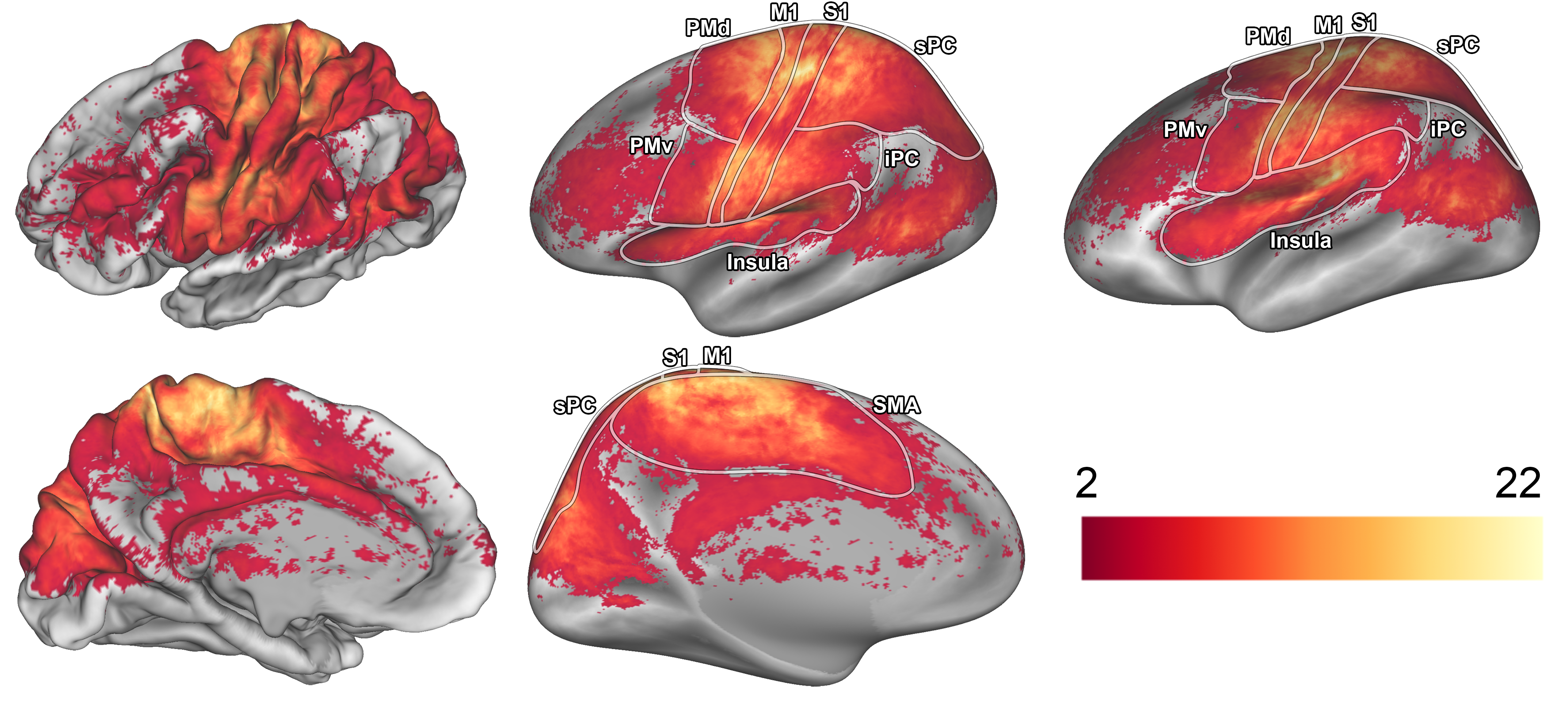

Supplement: S1 Fig — Goodness-of-fit F-statistic for the non-rigid pRF method is displayed on an average subject pial surface (left) and inflated surface (right) from a lateral point of view (top) and medial point of view (bottom). Hypothesis and error degrees of freedom for the calculation of the F-statistics were 19 and 837, respectively. The ROIs are denoted by the lines drawn on the surfaces: primary motor cortex (M1), primary somatosensory cortex (S1), supplementary motor area (SMA), dorsal premotor cortex (PMd), ventral premotor cortex (PMv), Insula/Sylvian fissure (Insula), inferior parital cortex (iPC), and superior parietal cortex (sPC). (TIF) [file pcbi.1009955.s001.tif]

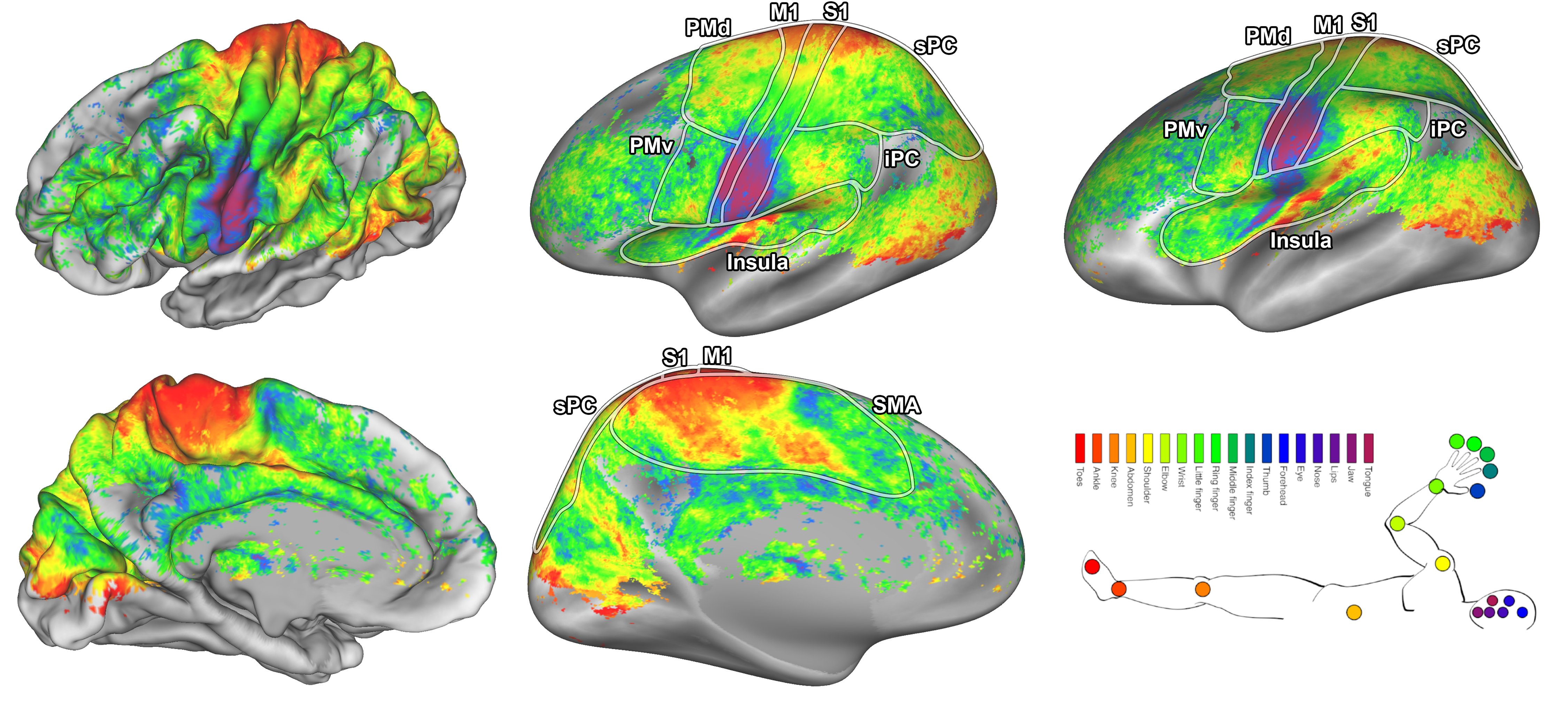

Supplement: S2 Fig — The conventional pRF centers are shown on the average subject pial surface (left) and inflated surface (right) from a lateral point of view (top) and medial point of view (bottom). Colors indicate the body part that was estimated as the pRF center. The ROIs are denoted by the lines drawn on the surfaces: primary motor cortex (M1), primary somatosensory cortex (S1), supplementary motor area (SMA), dorsal premotor cortex (PMd), ventral premotor cortex (PMv), Insula/Sylvian fissure (Insula), inferior parital cortex (iPC), and superior parietal cortex (sPC). (TIF) [file pcbi.1009955.s002.tif]

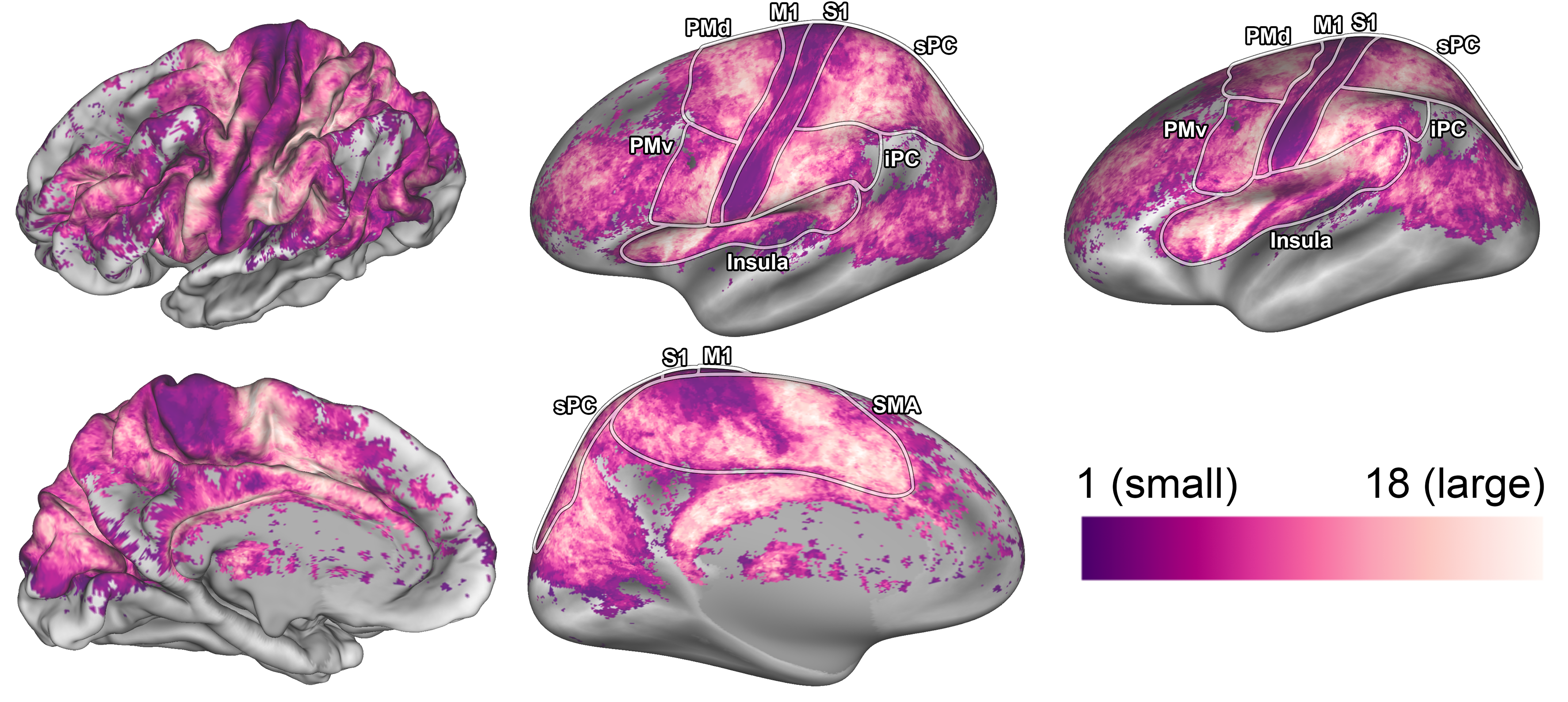

Supplement: S3 Fig — The conventional pRF size is shown on the average subject pial surface (left) and inflated surface (right) from a lateral point of view (top) and medial point of view (bottom). Colors indicate the pRF size. The ROIs are denoted by the lines drawn on the surfaces: primary motor cortex (M1), primary somatosensory cortex (S1), supplementary motor area (SMA), dorsal premotor cortex (PMd), ventral premotor cortex (PMv), Insula/Sylvian fissure (Insula), inferior parital cortex (iPC), and superior parietal cortex (sPC). (TIF) [file pcbi.1009955.s003.tif]

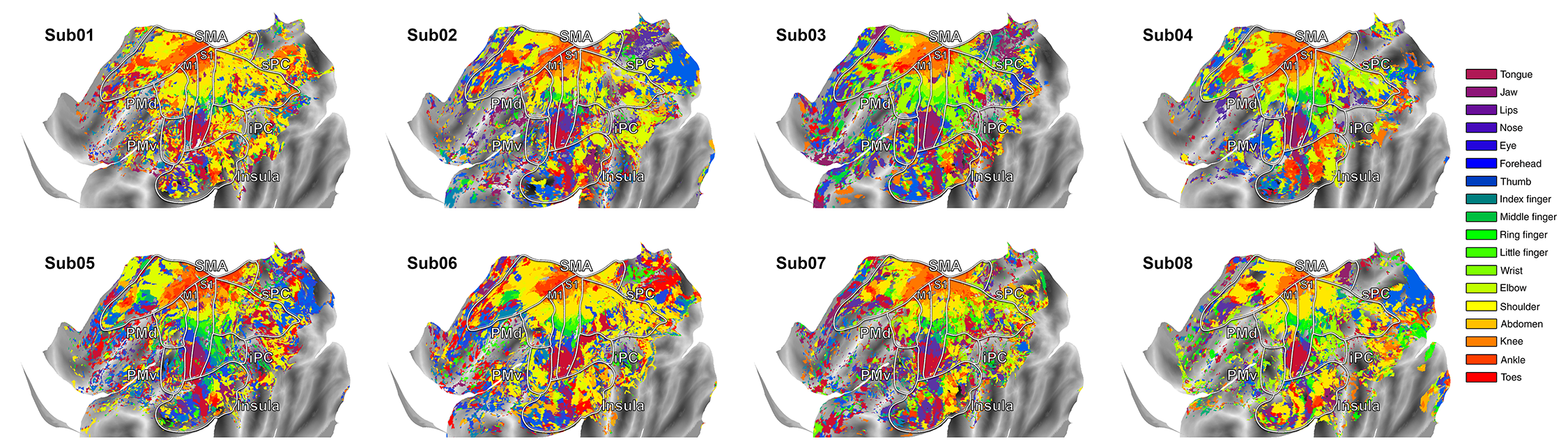

Supplement: S4 Fig — For each participant the non-rigid pRF centers projected on a flattened cortex reconstruction are shown with different colors representing different body parts. The ROIs are denoted by the white lines and text on top of the maps. (TIF) [file pcbi.1009955.s004.tif]

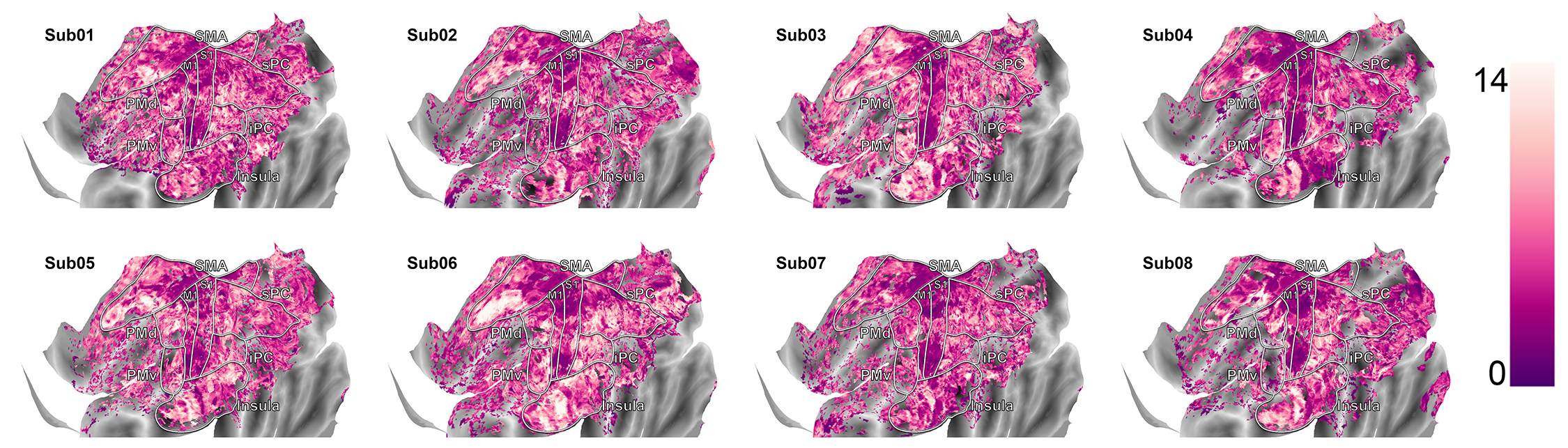

Supplement: S5 Fig — For each participant the non-rigid pRF sizes projected on a flattened cortex reconstruction are shown. Darker hues represent smaller pRF sizes and lighter hues represent larger pRF sizes. The ROIs are denoted by the white lines and text on top of the maps. (TIF) [file pcbi.1009955.s005.tif]

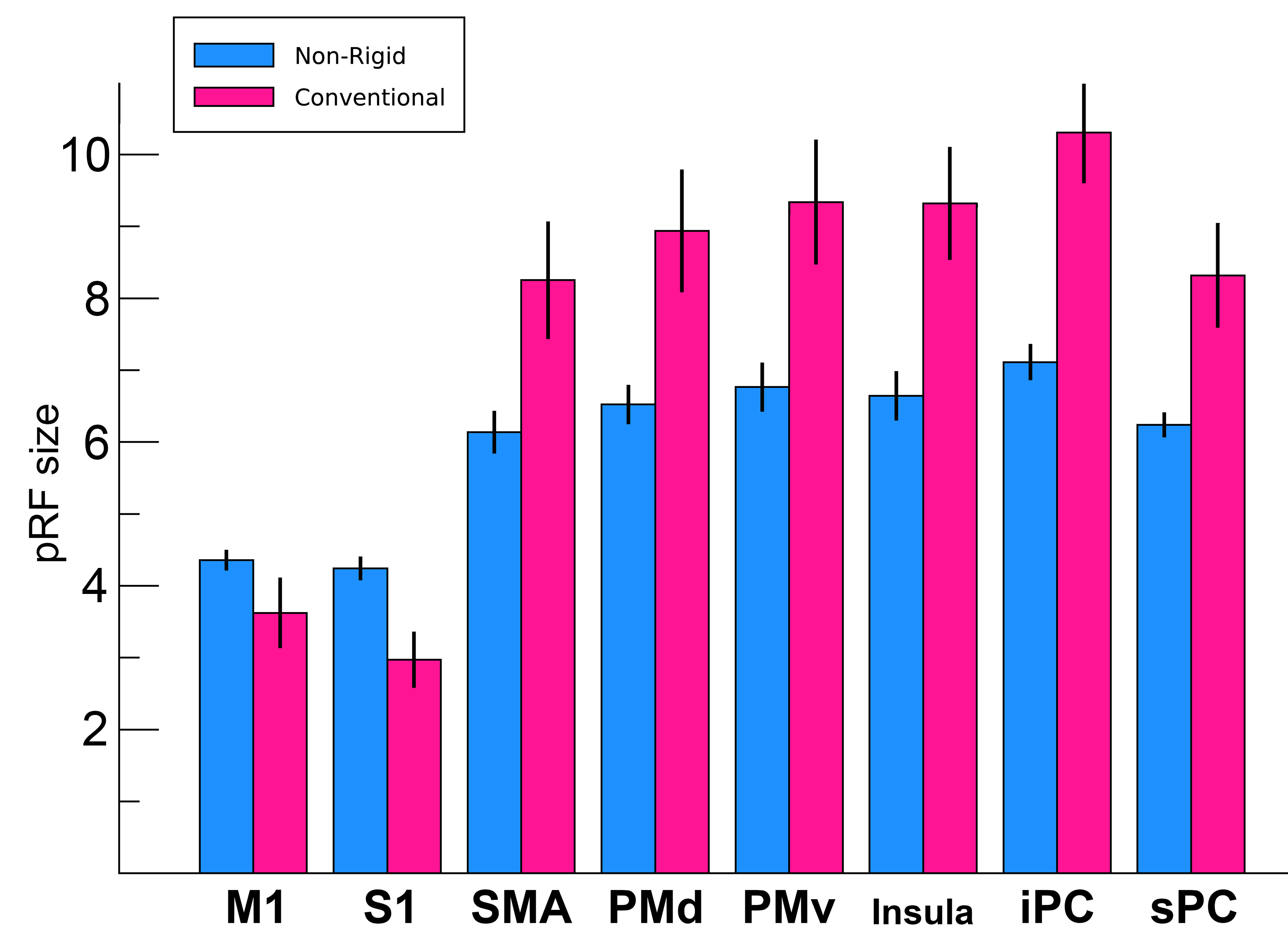

Supplement: S6 Fig — Comparison between non-rigid (blue) and conventional (pink) pRF sizes across ROIs. The conventional pRF model returns smaller pRF size estimates for areas M1 and S1, while the non-rigid pRF model returns smaller pRF size estimates for the other cortical areas. The error bars denote the S.E.M. across subjects. (TIF) [file pcbi.1009955.s006.tif]

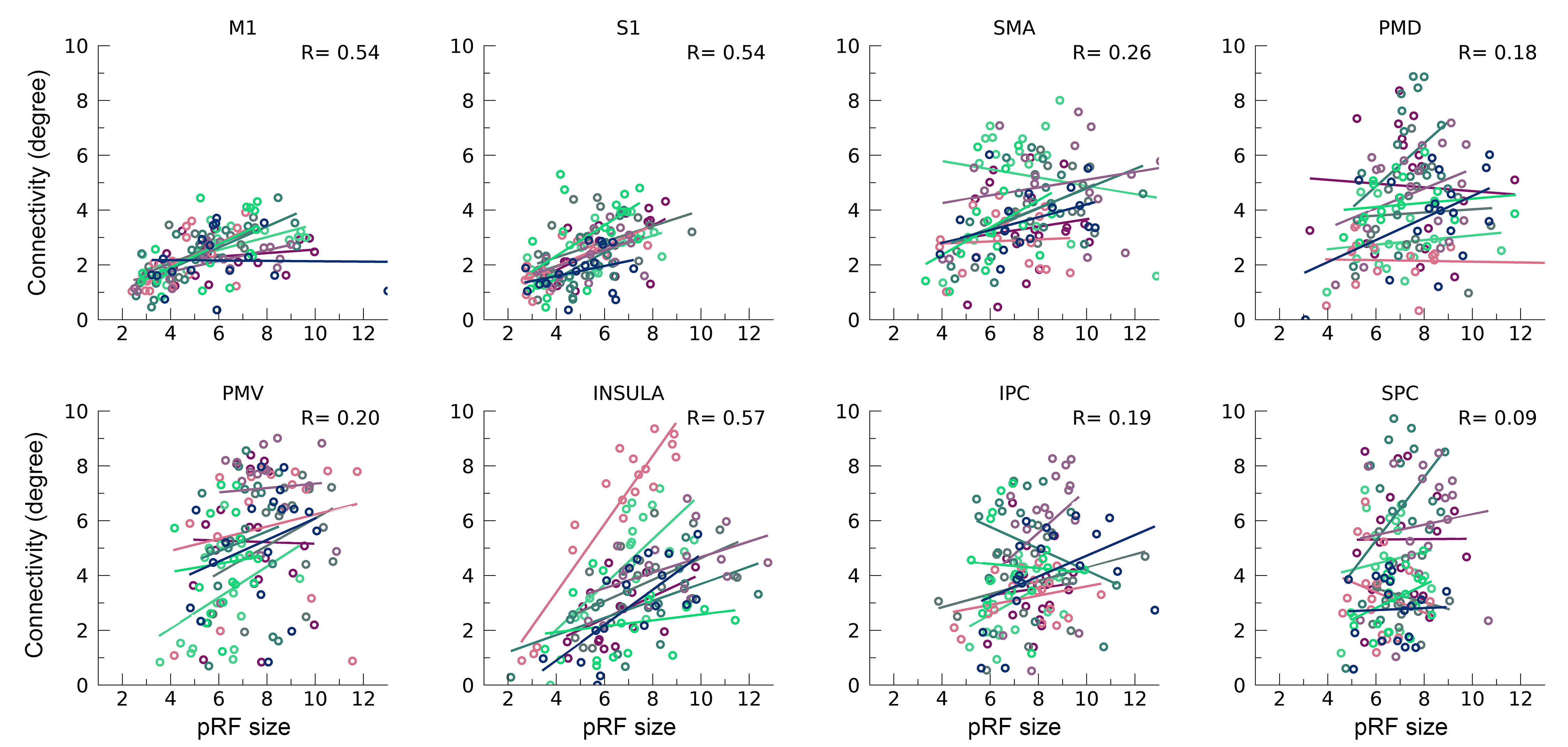

Supplement: S7 Fig — Each dot shows the relationship of pRF size (horizontal axis) with the Connectivity metric (vertical axis) of all 18 body part representations (individual dots) for each subject. The different colors correspond to the 8 subjects, for which the linear regression is shown by lines of the same color. Mean Pearson R across subjects for each ROI is presented in the top right corner of each plot. (TIF) [file pcbi.1009955.s007.tif]

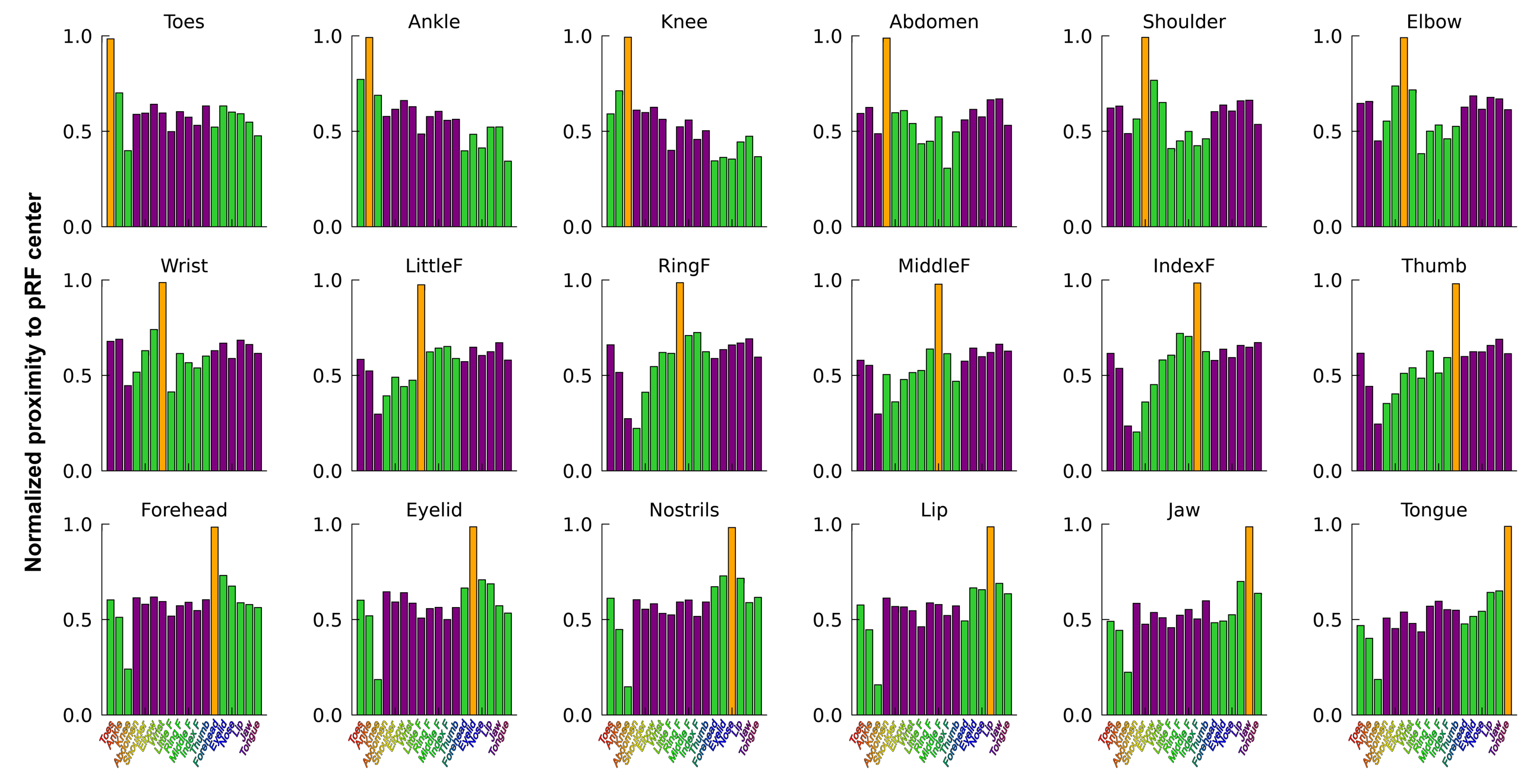

Supplement: S8 Fig — The normalized distances (1 = pRF center) for all cued body parts (horizontal axes) and all included surface vertices are shown, averaged over the estimated pRF center for each body part (separate panels). The estimated center body part is presented by the orange bar and by default is closest to the pRF center (i.e. highest normalized proximity value). The body parts that were presented during the same run as the estimated pRF center are represented by the green bars, while the body parts presented in the different run as the pRF center body part are shown by the purple bars. (TIF) [file pcbi.1009955.s008.tif]

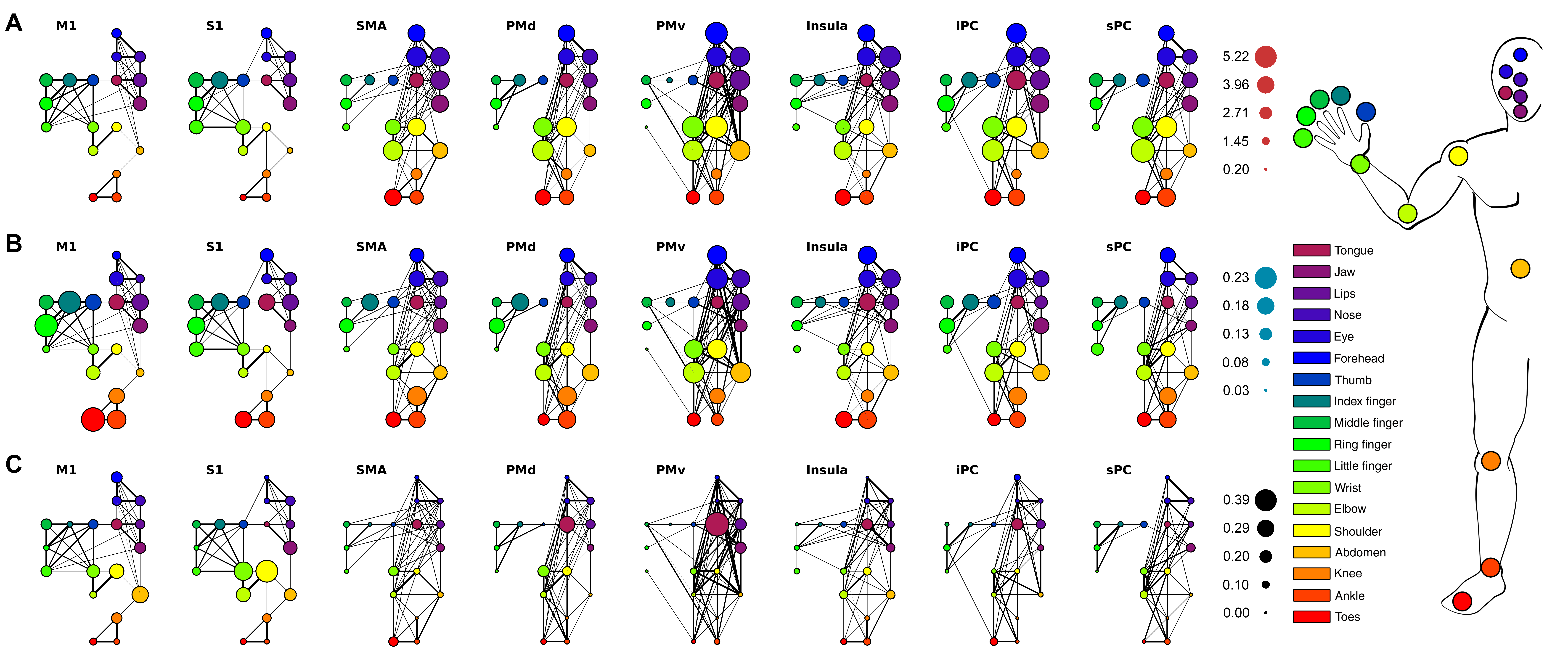

Supplement: S9 Fig — Whole-body-graphs are presented per ROI (from left to right) and for the connectivity, clustering and betweenness centrality coefficients (from top to bottom). The colors of each node in the graphs correspond to a specific body part given by the schematic at the far right. The number of surface vertices that contributed to each body part node was held equal. The connections between any 2 body part nodes was calculated per ROI and shown here through the lines connecting the nodes. The thicker the line the stronger the connection between body parts. (A) Connectivity values per body part node and ROI are depicted. The size of the body part nodes presents the size of the connectivity value per node. (B) Clustering coefficients have the same layout and graphs as the connectivity values. Here the size of the body part node reflects the strength of the clustering coefficient. (C) The size of the body part nodes in the ROI graphs reflects the strength of the betweenness centrality coefficient. (TIF) [file pcbi.1009955.s009.tif]

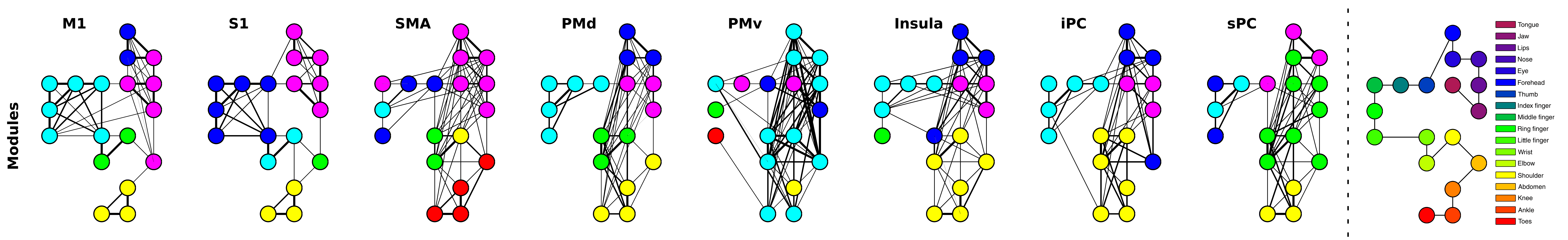

Supplement: S10 Fig — For each ROI, different modules are represented by different colors. Each module was determined on the basis of an equal number of surface vertices that contributed to the body part nodes. Note that the colors only define a cluster of nodes within one graph, and any correspondence of colors between graphs is purely accidental. The whole-body graph layout is presented at the outmost right indicating the node-body part relationship. (TIF) [file pcbi.1009955.s010.tif]
