# Supplementary material for: Moving in on human motor cortex. Characterizing the relationship between body parts with non-rigid population response fields
Source: PLoS Comput Biol. 2022 Apr 4;18(4):e1009955. doi: 10.1371/journal.pcbi.1009955 (PMC9009778; doi:10.1371/journal.pcbi.1009955)
Supplement: S1 Text — (DOCX) [file pcbi.1009955.s011.docx]

**S1 Text**

Since half of the body parts were cued in one run and the other half in the other run, we tested if estimated body part distances were unintentionally affected by task design. The non-rigid pRF analysis calculates 18 distances to a theoretical response field center (i.e. one for each body part) for each surface vertex. The pRF center body part is determined based on the shortest distance to the theoretical pRF center. In this particular test, we averaged the distances per pRF center (all vertices within the FOV), and then split the 18 distances in 2 groups: the first group represents 9 body part distances that were presented in the same run as the estimated pRF center body part, and the second group represents 9 distances of body parts presented during the other run relative to the pRF center body part. Using a Welch t-test we tested if the distances of body parts cued in the same run as the pRF center differ significantly from the distances of body parts cued in the other run. We found no evidence for a bias in distance estimation between body parts presented within the same versus the different run as the pRF center: Welch t_(13)_ = 0.342, p = 0.737. Apart from the knee (which suffered from relatively increased head motion), body part distances appear homogeneously distributed across the 2 different runs (S8 Fig).
