# Supplementary material for: Moving in on human motor cortex. Characterizing the relationship between body parts with non-rigid population response fields
Source: PLoS Comput Biol. 2022 Apr 4;18(4):e1009955. doi: 10.1371/journal.pcbi.1009955 (PMC9009778; doi:10.1371/journal.pcbi.1009955)
Supplement: S2 Text — (DOCX) [file pcbi.1009955.s012.docx]

**S2 Text**

In the main graph theory analysis, all significantly activated surface vertices were included in the analysis. The relationships between body parts were calculated on the basis of complete body part representations within each ROI. Hence, the cortical surface area that contributed to the graph theory analysis differed per body part. In this supplementary analysis, we calculated the same graph theory metrics as described in section 4.5, only here controlling for the number surface vertices per body part. For each subject and each ROI we determined the minimum number of vertices that allowed for the body part graph theory analysis to be based on an equal number of data points per body part. On average, approximately 81% (s.d. = 3%) of all significantly activated surface vertices were discarded to perform this “equi-representational” body part graph theory analysis. The graph theory metrics “connectivity”, “clustering coefficient”, and “betweenness centrality coefficient” were calculated as described in section 4.5, as was the qualitative measure for “Louvain modularity”. This procedure was repeated 10.000 times, each time randomly selecting a matched subset of vertices for each body part representation per subject and ROI. All graph theory metrics were averaged over the number of iterations afterwards, and were tested for statistical significance with the statistical analyses described in section 4.6.

Similar to the main analysis, the equi-representational graph theory analysis indicates that connectivity values differ significantly across body parts (F_(17,119)_ = 4.38, p < 0.001) and cortical areas (F_(7,49)_ = 9.90, p < 0.001, S9A Fig). Both the knee and the little finger were less connected within the graphs compared to other body parts (t_(119)_ = -3.72, p = 0.001, t_(119)_ = -4.83, p < 0.001, respectively). The connectivity averages of cortical areas reveal that body parts in M1 and S1 are less interconnected (t_(49)_ = -5.02, p < 0.001 and t_(49)_ = -4.37, p < 0.001, respectively). Contrary to the main analysis, area PMv does not display significantly larger connectivity values as was reported in the main analysis (t_(49)_ = 1.84, p = 0.072), whereas area iPC does show significantly larger connectivity values (t_(49)_ = 4.60, p < 0.001). Similar to the main analysis, the clustering coefficient differed across body parts (F_(17,119)_ = 4.46, p < 0.001), although the ring finger does not exhibit significantly larger clustering coefficients (t_(119)_ = 0.28, p = 0.783), while relative larger clustering coefficients were found for the ankle (t_(119)_ = 2.57, p = 0.011). No clustering effects were observed across cortical areas (F_(7,49)_ = 1.02, p = 0.426, S9B Fig). Finally, we found that betweenness centrality coefficients differ across body parts and cortical areas (F_(17,119)_ = 4.05, p < 0.002 and F_(17,49)_ = 11.95, p < 0.001, respectively), which was also observed in the main analysis. Similarly, the shoulder and the wrist exhibited larger betweenness centrality coefficients compared to all other body parts (t_(119)_ = 2.75, p = 0.007 and t_(119)_ = 2.75, p = 0.007, respectively). In the equi-representational analysis, the tongue additionally displayed a significantly larger betweenness centrality coefficient (t_(119)_ = 4.85, p < 0.001). Additionally, body parts in M1 and S1 contain on average larger centrality coefficients (t_(49)_ = 6.13, p < 0.001 and (t_(49)_ = 5.43, p < 0.001), while average centrality coefficients in PMv are not significantly smaller (t_(49)_ = -1.76, p = 0.085), but betweenness centrality coefficients in sPC are significantly smaller compared to other areas t_(49)_ = -3.02, p = 0.004, S9C Fig). Finally, Louvain modularity revealed body part modules in the equi-representational that are comparable to the modules derived in main analysis (S10 Fig).
