# Supplementary material for: Moving in on human motor cortex. Characterizing the relationship between body parts with non-rigid population response fields
Source: PLoS Comput Biol. 2022 Apr 4;18(4):e1009955. doi: 10.1371/journal.pcbi.1009955 (PMC9009778; doi:10.1371/journal.pcbi.1009955)
Supplement: Attachment — Submitted filename: Rebuttal.pdf [file pcbi.1009955.s014.pdf]

<u>Reviewer #1:</u>

This paper makes two novel methodological contributions. 1) They use a 'non-rigid' model to estimate a motor control population response field (pRF) in single fMRI voxels. 2) They apply graph theoretic measures to analyze the relationships between the pRFs in different brain regions. The non-rigid pRF method is an interesting proposal that could have applications beyond the study of motor control. As for the empirical results, my expertise is in modeling the visual system, so my judgment of their novelty is largely based on the author's descriptions and citations. These seem to indicate that the results are in agreement with prior work, with some interesting results but no big surprises or major theoretical advances.

I believe this paper would be suitable to publish at PLOS Computational Biology with major revisions.

The first major point of revision is in interpreting the graph theoretic measures. In the abstract and the introduction, the authors assign each formally defined measure an interpretative term (i.e. connectivity = 'uniqueness', clustering = 'clique-formation', and betweenness centrality = 'importance'). I think this is a useful way to convey the results. However, I think the authors should reconsider or reframe these terms for clustering and betweenness centrality. The weighted clustering coefficient only examines cliques in triangles around some node, which means that it cannot capture any higher-order cliques. Higher-order cliques may actually capture some interesting behavior in motor control regions. One reference about higher-order cliques can be found here: https://www.cs.cornell.edu/~arb/papers/higher-order-clustering-PRE-2018.pdf The authors should either address this limitation in the interpretation, or explore the results with some higher order clustering coefficients.

We thank the reviewer for this comment and acknowledge that the clustering coefficient used in our manuscript only describes simple clique formations. In the current manuscript, we chose to apply commonly used graph-theory metrics to avoid overcomplicating our analyses and subsequent interpretations. For that reason, we chose not to include a higher-order clustering analysis, as we feel it will not benefit the interpretation of clique-formation. We now discuss this issue at L393-395 including the statement that higher-order clustering might contain additional information about the relationship between body part representations.

Additionally I think describing betweenness centrality as 'importance' can be misleading, or at least raises many questions. Important for what? More important for the brain to represent in high fidelity? Important for overall motor control? etc. I accept the author's extended definition in the introduction, as "the (indirect) involvement of a particular body part when other body part moves" (lines 103-104). I think another appropriate description would be that the betweenness centrality measure describes how much the representation of the motion of a single body part overlaps with the motion of other body parts. My best suggestion is to replace 'importance' with something like 'representational centrality' or 'representational overlap'. Either way, descriptions of this measure throughout the Discussion should be revised with this point in mind.

We understand the reviewer's comment that our definition of betweenness centrality can lead to unclear interpretations. The aim was to present a definition of betweenness centrality in relation to body part movements without using circular terms (i.e. without using the terms 'betweenness' or 'centrality'). We thank the reviewer for his/her constructive suggestions, but for the reason mentioned earlier, we cannot accept the term 'representational centrality'. Also

the term ‘representational overlap’ does not adequately reflect the meaning of betweenness centrality, as betweenness centrality does not merely reflect the overlap of response fields (which is more or less captured by the ‘degree’). Therefore, we decided to go with another option: ‘body part influence’. We think that ‘body part influence’ adequately and fairly unambiguously explains betweenness centrality as the relatively increased influence of a particular body part during the movements of other body parts.

The second major point is a possible error with the manuscript or data. In Figure 6, the authors show graphs for each ROI and graph theoretic measure, where the node sizes correspond to the value of each measure. From my understanding, the edges of the graph should simply be derived from the correlation matrix of the average response fields, which is done separately for each ROI. This implies that the graph edges should be identical in the 3 graphs of each column of Figure 6, but that is not the case. For example, in 6A S1 there is an edge from the abdomen to the knee, but 6B S1 does not have this edge. Why is this the case?

This issue was detected by the authors as well and is solely caused by Microsoft Word & Adobe Acrobat, which were used to create the manuscript and pdf-file. In the original figure, all edges within the graph of a cortical region are exactly the same for Figures 6 A, B & C. To minimize the chance that the final published figure suffers from similar defects, the figure has been updated with broader lines (which has now become Figure 7).

Other miscellaneous points:
- The authors find evidence of somatotopy in all ROIs. However, Fig. 2 suggests that not all regions contain complete representations of the 18 areas (e.g. PMv appears to missing red ankle/toe activation). It would be useful to investigate this, or at least show a more complete plot of the evidence for somatotopy. For each ROI, the authors could plot the x/y coordinates of each voxel (each point colored with the body part preference) with the somatotopy regression line plotted on top.

Figure 5 (previously Figure 4) shows the mean cortical coordinates per body part, per cortical area. The cortical coordinates were rotated, so that any somatotopic structure should be present along the vertical axis, which renders the other (horizontal) axis redundant. The assessment of somatotopic structures was based on this data. Plotting the X/Y coordinates for each voxel of each subject does unfortunately not lead to a clearer figures.

However, the reviewer touches upon an important aspect of assessing the presence of a somatotopic structure: How many voxels or surface vertices at minimum should contribute to the representation of a single body part, and how much body part representations are needed to conclude a somatotopic organization? We do not know the answers to these questions, as many different configurations could still be considered evidence for a somatotopic organization (e.g. some body parts not represented, some body parts represented at the ‘wrong’ location, or some body parts represented by only 1 voxel at the expected location). However, we added a figure showing the surface area of individual body part representations (new Figure 2) in addition to the already existing body part coordinates in Figure 5. The surface area is a better measure than a scatterplot or histogram of voxels or surface vertices representing body parts, since it accounts for surface curvature. This new Figure 2 can be used to assess the evidence for body part representation, whereas Figure 5 can be used to assess the evidence for somatotopic organizations. This topic is now also discussed in the manuscript at line L164-165 & L346-349.

- Is there some systematic relationship between pRF size and any of the graph theoretic measures? This might be useful to further illustrate what the various graph theory measures add beyond the traditional pRF approach.

We thank the reviewer for this remark. There appears to be a relationship between pRF size and the connectivity metric (degree) (mean Pearson R=.32, see Figure 1 below), but not between pRF size and betweenness centrality (mean Pearson R=.05) or the clustering coefficient (mean Pearson R=.04). The relationship between pRF size and connectivity is not unexpected: the pRF size was calculated as the number and spread of body part representations per cortical surface mesh vertex, which was grouped afterwards on the basis of pRF centers to perform statistics. The connectivity metric, however, is based on the full response field profiles, which were averaged over surface vertices with the same pRF center, creating mean pRF profiles. These mean pRF profiles per body part were then correlated with each other to create the whole-body graphs per cortical area. The number of node connections within the graphs represents the connectivity metric (degree).

Thus, both statistical analysis performed on pRF size and connectivity are based on pRF profiles of vertices that share a common pRF center. However, the pRF size is calculated for each individual vertex (or voxel) separately, and does not contain information on the relatedness to vertices of a different pRF center, unlike the connectivity metric. This differences also applies between pRF center and clustering coefficient or betweenness centrality coefficient. The more homogenous response profiles are of individual vertices with the same pRF center, the more correlated pRF size and connectivity are likely to be. We added clarifications to the manuscript at lines L384-387

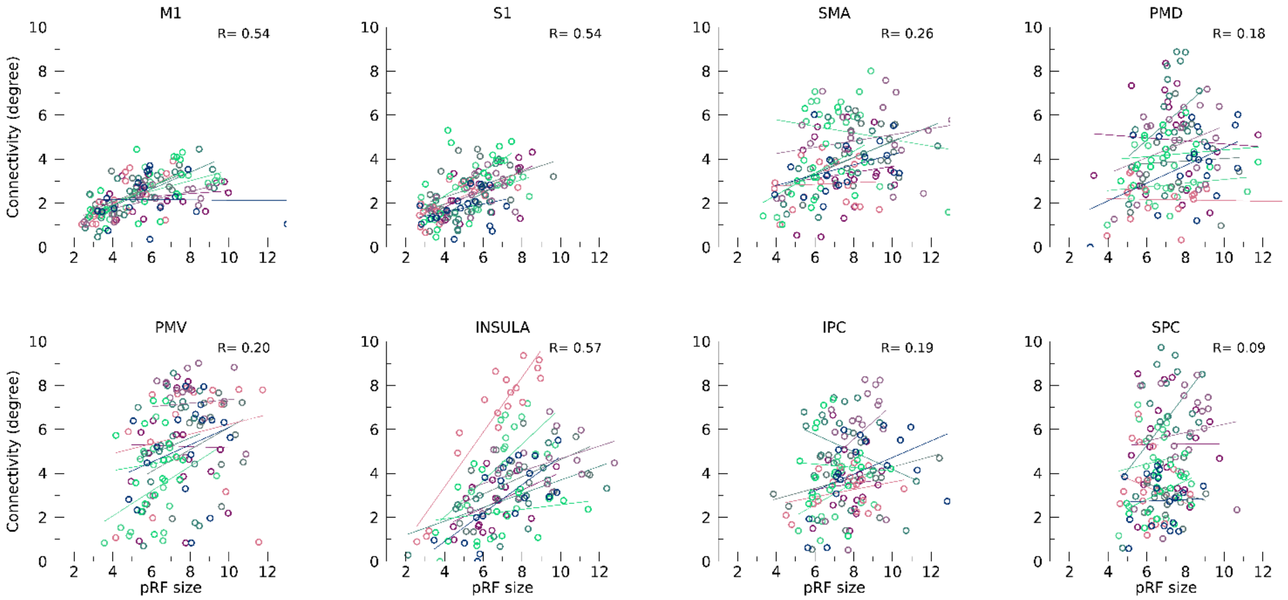

Figure 1. Relationship pRF size and Connectivity (degree). Each dot shows the relationship of pRF size (horizontal axis) and Connectivity metric (vertical axis) of all 18 body part representations for each subject. The different colors correspond to the 8 subjects, for which the linear regression is shown by lines of the same color. Mean Pearson R across subjects for each ROI is presented in the top right corner of each plot.

- Is there any substantial intersubject variation (particularly for the results in Fig 6)? Along the same lines, it would be nice to see individual points for each subject in Fig 5 instead of / in addition to the SEM error bars.

Yes, there is intersubject variation. Whether or not this is substantial is not an easy question to answer. It is important to note, that the vast majority of statistics is calculated across subjects. Thus, the reported findings take subject variation into account. Intersubject variation also differs across cortical areas, being relatively smaller in M1 and S1 compared to the other areas. The standard deviation (S.D.) across subjects of the mean response profiles in primary sensorimotor cortex is: mean S.D. M1 & S1 = 0.12, while mean S.D of the other areas is 0.16. Since the original values range between 0 and 1, these S.D. values can be interpreted as percentage variation of the maximum variation across subjects.

To be able to review the intersubject variation, we offer an alternative to Figure 6 with points for individual subjects (Figure 6b). Personally, we feel that the Figure 6b is less comprehensible than the original Figure 6. The original Figure 6 is already fairly data-rich, and we feel that Figure 6b is too cluttered. Hence, we propose to keep the original Figure 6.

- Is it possible that the results for voxels preferring some part of the head (body parts 13-18) are affected by a confound with head motion and the fMRI data? Even after motion correction, large movements can cause biases in the fMRI data. How much motion do subjects typically exhibit, and were there outliers to exclude?

It is a well-known fact that head motion causes motion artefacts in fMRI measurements that cannot be completely corrected for with scan realignment. It is, however, not clear how motion artefacts affect pRF metrics, and whether particular body part movements cause more head motion than others. To investigate this, we summed the total estimated head motion during the first 3 seconds after any motion cue onset. The summed total estimated head motion, was calculated as the sum of the first derivative of all 6 rigid body motion parameters squared. This procedure results in positive values (of an arbitrary unit, as this is a combination of translation and rotation first derivatives) for any rigid body head movement relative to the previous time point (i.e. scan). We find that only the knee movement caused significantly larger head movements (Knee: Welch $t_{(7)}=3.18$, $p=0.015$, see also Figure 2 below). Plausibly, extension-flexion of the knee, while lying on an MRI-table bed, causes the entire body to shift. It is possible that increased head movement during knee movements may be related to the relative smaller pRF sizes for cortical knee representations. Interestingly, the cued head body part movements did not result in significantly larger estimated head motion (Welch $t_{(110)}=-1.85$, $p=0.067$) The finger movements, however, resulted in significantly less estimated head motion (Welch $t_{(103)}=-3.46$, $p<0.001$). We choose not to exclude any participants or body part movement conditions from the results, because we want to represent the data in full “as is”. The additional head movement estimation presented here is also included in the manuscript and possible effects on pRF estimates is discussed in L143-147 & L380-382

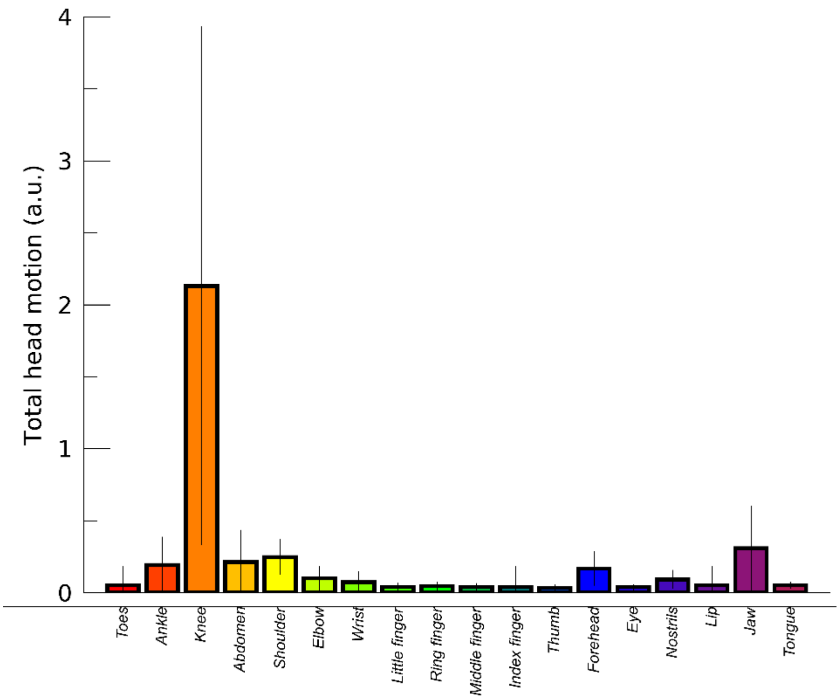

Figure 2. Estimated total head motion. Head motion per body part cue, estimated as the sum of the first derivative of the 6 rigid body motion parameters (0-3s after motion cue onset).

- While I do not believe it will change the conclusions of this report, it would be nice to see some examination of the distribution and sphericity assumptions of the ANOVA data. Some sphericity correction may be needed.

For all performed repeated measures ANOVA analyses the sphericity assumption for the cortical areas condition was not violated. However, there were not enough degrees of freedom to perform Mauchly's test of sphericity on the body parts condition, nor on the interaction of the body parts and cortical areas conditions.

<u>Reviewer #2:</u>

*In the manuscript "**Moving in on human motor cortex. Characterizing the relationship between body parts with non-rigid population Response Fields**", Schellekens and colleagues investigate the relationship between body part representations in sensorimotor cortices, using a non-rigid population Response Field (pRF) method that they developed, combined with graph theory. From the pRF mapping, the authors report somatotopic organisation in all included motor areas, and a higher selectivity in primary cortices than in secondary ones. Applying graph theory on this data, they report unique response profiles for the knee, that digits of the hand cluster around the ring finger and a central role for the shoulder and elbow. The authors conclude that these results reveal associations among body parts at the cortical level that are in agreement with intuitive notions of body part usage. Finally, the authors emphasise that combined with graph theory, their new pRF method allows to investigate neuronal population response fields, when the relationship among selected functions is not apriori known.*

The study is well-designed, well-written and using cutting-edge techniques to investigate a very interesting question that has not been addressed to my knowledge. However, some methodological aspects make it difficult to understand what can be gained from all these

impressive analyses, apart from the new pRF method they describe and validate using the conventional pRF method. Here are my major comments:

<u>Related to the design:</u>

- lines 506-507: 'The functional session was split in 2 runs (torso/arm/hand and head/leg, figure 1)'.

It seems to me the authors cannot directly compare in their analyses the data from the leg/face and the hand/trunk since it was acquired in different runs. The authors should have presented all conditions in each run, to avoid introducing a bias both in the pseudo-randomisation of the conditions (not fully pseudo-randomised here), and in the run-dependent variability/noise introduced in the design. Rescaling to percent BOLD signal change does not fully account for that.

We thank the reviewer for the opportunity to explain our experimental choices. We chose to split the conditions in a hand/trunk and a leg/face part to avoid 'missed cues' by the participants. Having participants focus on a whole body image with 18 possible movement cues, would inevitable have resulted in missed cues, and additional head/eye movements, caused by the continuous scanning for movement instructions. The cues would have to have been smaller, and participants might be fixating at the 'wrong' location of the visual field. Therefore, we chose to split the cues in 2 parts, allowing for larger cues and a more centralized presentation with respect to the center of the screen in the scanner. However, regardless of our reasons for the experimental design, this cannot be undone. The question that remains, is whether or not this is a problem. We calculated the tSNR (temporal Signal-to-Noise) ratio after the sessions were preprocessed, high-pass filtered and converted to percent signal change. Using a repeated measures ANOVA with one factor for the different runs, we find that there is no evidence for a difference in tSNR across the different runs ($F_{(3,18)}$=0.18, p=.910). Thus, there is no reason to assume that we insufficiently accounted for the fact that the task was presented across multiple runs.

- lines 205-207: 'We ensured that the movement order of body parts during scanning was randomized to prevent body parts from being coupled to one another on the basis of the experimental design.'

I understood from the lines 506-507 cited above that the torso/arm/hand conditions were tested in one run and the head/leg conditions in another. If I misunderstood, please rephrase lines 506-507 because it is misleading this way. If I understood correctly, please rephrase lines 205-207 to make explicit that all conditions were not fully counter-balanced and that as a result some body parts might be more coupled than other (e.g., leg/head might be artificially more coupled than leg/hand).

Lines 224-226 have been rephrased to: We ensured that the movement order of body parts within a single scan acquisition run was randomized to prevent body parts being coupled to one another on the basis of the experimental design'

Thus, this means that the design prevented body parts to be coupled within a single run, and please note, that body parts are naturally decoupled between different runs.

<u>Related to the new pRF method:</u>

- If the conventional approach gives similar maps and pRF sizes, could the authors explain what is the point of this non-linear approach, at least for the sensorimotor regions?

Thanks to the reviewer for this opportunity to clarify the relevance of our newly developed pRF method. We developed to the non-rigid pRF method, because we wanted to avoid making assumptions on the internal pRF structure *a priori*. This also makes the non-rigid pRF method applicable to all sorts of tasks. Interestingly, the non-rigid pRF shows that assuming an 'homunculus' internal pRF structure in primary sensorimotor cortex is on average a valid assumption. Nevertheless, we would not have known this without the novel non-rigid pRF method, nor was there sufficient evidence to assume it beforehand. Additionally, an internal homunculus pRF structure may not hold for every voxel or specific regions of cortex. Thus, the point of the novel non-rigid pRF method is to extract pRF properties without making *a priori* assumptions on the internal structure of neuronal populations, and the ability to further evaluate the estimated internal pRF structure (see also manuscript L78-81). We, additionally added further clarifications at L458-460

- The authors should take a control ROI that is not sensorimotor to make sure the method is not introducing any biases.

Unfortunately, it is not clear to us what sort of bias the reviewer is referring to. Hence, it is not clear what sort of control test is needed to assess such bias. Also, we are not sure which control region would be selected. Virtually every cortical area within the FOV is involved in motor (task) perception/initiation/execution/monitoring. Even though some regions of cortex do not exceed the significance threshold, this does not mean that those regions are devoid of motor task induced BOLD responses.

- The authors have not tested all possible body parts, so by selecting the lowest distance to the center, the authors may have misassigned pRF centers. Indeed, if the lowest distance exceeds a certain distance, the vertex should not be assigned a pRF center. The authors could for instance use the goodness of fit to select which vertices to keep for the pRF mapping and the related measures.

We thank the reviewer for these remarks and suggestions. We already used the goodness-of-fit statistic for vertex selection, which now more clearly mentioned at L727-729. However, the reviewer is correct: unless you test every possible thing that the human brain could represent or be involved in, one can never be sure that the estimated pRF center is the 'true' pRF center for a given neuronal population. This is also true for visual cortex pRF mapping, where the pRF center is subject to stimulus presentation. In any pRF analysis regardless of the modality, pRF center & size are relative to the experimental conditions. Nevertheless, the reviewer raises an important issue, whether we should exclude vertices on the basis of minimum center distance exceeding some threshold. Over 90% of all vertices of all subjects staid within the original 1/5th of the half-width-at-half-maximum (i.e. FWHM/2) cut-off (see Figure 3 below). It is not clear where and why an additional distance-defined cut-off should be imposed. Hence, we did not choose to exclude vertices that have a minimum distance larger than zero and smaller than the original FWHM/2 cut-off.

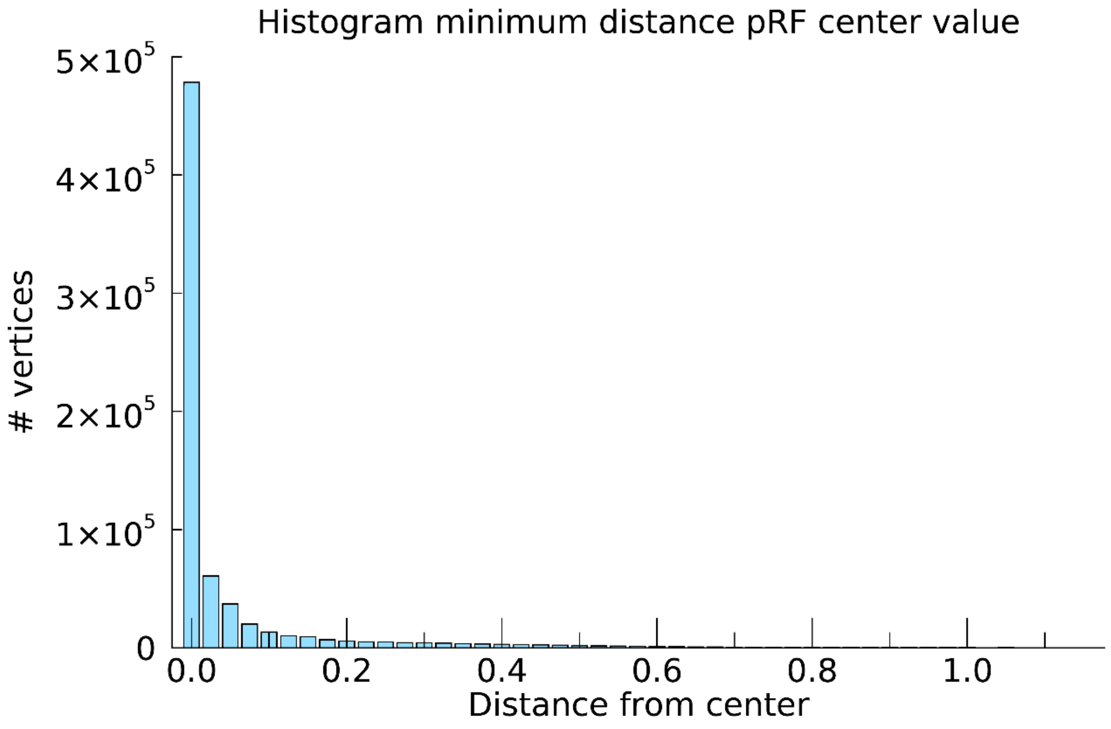

Figure 3. Histogram minimum distance pRF center value. The histogram includes all selected vertices of all participants. The original cut-off is imposed at a distance of FWHM/2 ≈1.17

- The mean and range of goodness of fit obtained across all vertices and ROIs should be reported.

We thank the reviewer for this suggestion. Rather than presenting the goodness-of-fit F-statistic, we are currently showing the more intuitive variance explained by the pRF fit on the data by means of R^2-values for each region (including standard deviation). These values are currently reported at lines L148-153. Supplementary figure 3 does show the goodness-of-fit F-statistics displayed on the cortical surface meshes.

- Can the authors explain better what they mean line 579-581: 'In case of multiple body parts with the lowest distance to x0, the pRF center was calculated as the mean index of the multiple body parts and rounded to the nearest whole integer'. I understand that if for instance body parts 1, 6 and 8 (e.g., toes, elbow, little finger) ended up having the same lowest distance to x0, the authors took the mean of their indexes (here 5), and assigned that vertex to body part 5, which is the shoulder. If I understood correctly, this is inserting a lot of biases and assuming only neighbouring body parts would have such conflicting issues. It may artificially make the distances and pRF maps more linear/smooth.

We are content to conclude that the reviewer understood correctly. However, this was a choice that needed to be made with respect to vertices, where multiple body parts were estimated at the center of the response field. Particularly, the display of pRF centers on the cortical surfaces required a single value. Alternative solutions have downsides as well: e.g., center of mass/gravity biases towards the middle of the homunculus, taking the first, middle or median

body part disregards the other body parts, and our solution smooths across the multiple center body parts. However, the percentage of vertices to which this procedure applied was less than 1% for each participant (average percentage of vertices with more than 1 center body part = 0.77%, S.D. = 0.07%, now also mentioned at L165-166). Therefore, we argue that this is an acceptable solution with negligible consequences, and that more than 99% of all presented values are not affected by its downsides.

- The fact that the authors report large pRF sizes for the fingers also in S1 is counter-intuitive, both in terms of receptive size and in terms of selectivity. Finger representations in S1 are known to be quite selective and to display the smallest receptive fields.

We thank the reviewer for this chance to elaborate on our pRF findings. We agree with the reviewer that it sounds completely intuitive to assume smaller pRF sizes for cortical finger representations, especially in S1. However, there are 2 particular effects/topics to consider here. First and foremost, this is motor induced activity. In other words, the activity observed in S1, might not be fully driven by somatosensory (proprioceptive) input. It has been reported that M1 projections influence S1 activity through infra- and supra-granular connections during motor tasks (e.g. see Adesnik & Naka, *Cracking the function of layers in sensory cortex*, 2018). This may very well influence (increase) the pRF sizes observed in S1. Possibly, the effect of larger pRF sizes in S1 during motor compared to somatosensory tasks can be observed by comparing our previous articles: Schellekens et al., *Detailed somatotopy in primary motor and somatosensory cortex revealed by Gaussian population Receptive fields*, 2018 and Schellekens et al., *A touch of hierarchy. Population Receptive Fields reveal fingertip integration in Brodmann areas in human primary somatosensory cortex, 2021*. In these articles, the unit of pRF size is comparable, showing that pRF sizes during somatosensation in S1 are almost half the size compared to S1 pRF sizes during finger movements. A second important aspect to consider is that -to our knowledge- there are currently no studies that quantify receptive fields in S1, while including body parts across multiple limbs or even the whole body. It is impossible to compare receptive field sizes between e.g., finger representations, face representations, or lower limb representations if the different sets of body parts are not included during the assessment of receptive field size. Thus, it may sound intuitive that finger representations should have smaller receptive fields than other body parts, however, there exists no substantial evidence to support this claim (at least not for motor activity). We now discuss this topic in the manuscript at lines L369-371.

To understand a bit better and compare to pre-existing results, it would be interesting to report the inter-finger data. Do the authors see something similar to what was reported by Puckett et al, 2020? Do they observe different pRF size across fingers? Do they observe different pRF size within SI (along the antero-posterior axis)? It would be interesting to include BA2 in the S1 ROI to compare the different subregions (i.e., BA3b,1,2). In this regard, the conventional approach seems to give more consistent pRF sizes, with the smallest pRF sizes located in S1 and it seems more in BA3b,1 than in BA2. The authors should discuss the results observed in S1.

We thank the reviewer for these constructive suggestions, however, we disagree on this topic. The main reason for us not to separate S1 into its respective Brodmann areas is that this study concerns motor-induced activity. Hence, the current results are not directly comparable to Puckett et al., 2020. The results are not even fully comparable to our previous finger movement experiment (Schellekens et al., 2018) because here we combined a flexion-extension movements in one cue, whereas we separated these movements in our aforementioned 2018

study. If the reviewer is interested in pRF properties for finger movements in S1, we would suggest that our aforementioned 2018 study offers a good perspective.

Since the manuscript presents a motor experiment, there are more logical ROI candidates to be split up (e.g., M1 -> BA4a & BA4p, or SMA -> pre-SMA & SMA-proper). However, there is an important reason not to split these areas up: certain ROIs may become too small, which may lead to certain body parts being underrepresented or not represented at all. This will hinder statistical comparisons. Additionally, the number of vertices per ROI will affect and possibly bias the statistical tests when comparing between cortical areas. Therefore, we choose to present our non-rigid pRF results across relatively larger functionally distinct cortical areas.

- lines 594-597: 'Since body parts were only fitted in one side of the Gaussian shaped response field, only body parts that were in range of half the width at half maximum were included in the pRF size calculation'.

It is not clear which body parts we included/kept for the pRF size measure. The fact that some body parts were not taken into account should be reported in the main text, as well as which body parts were in the end used for that measure in each ROI.

We thank the reviewer for this opportunity to clarify the pRF procedure. We understand that these specific sentences from the manuscript may cause confusion. What is meant is the following: the static response field shape was that of 'half of a Gaussian' with $\sigma=1$. This way we do not have to make assumptions about a left/right-hand side relationship of body parts relative to the center. We then define the pRF size -similar to the conventional pRF method- in direct relationship to the Gaussian standard deviation (σ). In the conventional pRF method, this Gaussian σ is actually one of the parameters to be fitted, while in the non-rigid pRF method this is stationary. Nevertheless, we can still assess the spread of fitted body parts within the range of this static Gaussian σ, which is logically related to the FWHM ($FWHM=2*sqrt(2*ln(2))*\sigma$). However, because we fit body parts in 'half of a Gaussian', we consider the spread of fitted body parts within the FWHM/2 (or: the half width at half maximum). In other words, nothing is discarded, but this is simply the way to calculate the non-rigid pRF size that remains closest to the conventional pRF method. We have clarified this in the manuscript at L637-639.

- The mention 'although pRF sizes estimated by the conventional pRF method tend to be larger on average' is not informative to me and even misleading since the conventional approach seems to result in smaller pRF sizes for S1, M1 and the insula, and larger sizes for the other ROIs.

The reviewer is correct: the conventional pRF sizes are smaller in M1 and S1, but larger everywhere else (Figure 4 below). We have adjusted this in the manuscript (L204-205).

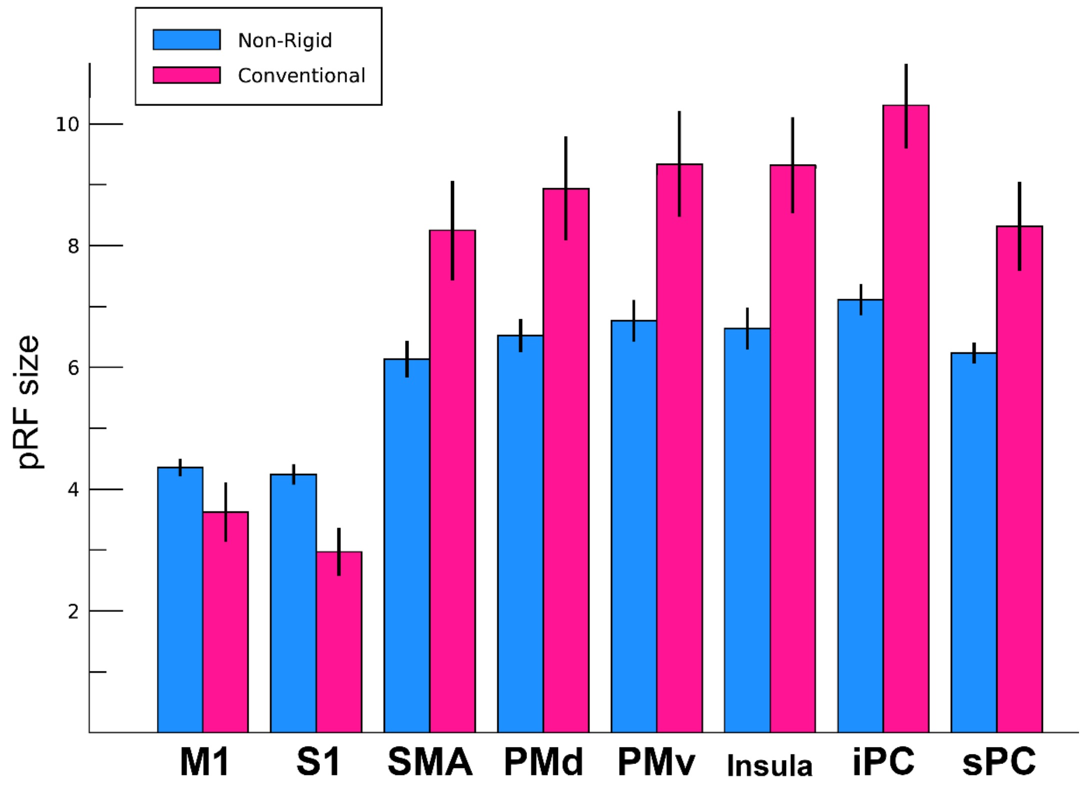

Figure 4. pRF sizes. Mean pRF sizes per cortical area and pRF method (colors). Error bars represent the SEM across subjects.

<u>**Related to the graph theory analysis:**</u>

- Graph theory requires nodes to be independent from each other (i.e., to correspond to independent structures or representations) and of the same size (i.e., same number of voxels) to avoid biasing the results towards one node or another. The way nodes are defined is highly affecting the outcome measures, both quantitatively and qualitatively (see e.g., Zalesky et al, 2010; Fornito, Zalesky & Bullmore, 2010; Hayasaka & Lauriento, 2010; Wang et al, 2009). Here the authors selected the vertices showing significant goodness of fit for each body parts (lines 611-613), but this is likely to results in different number of vertices. And care should be taken to avoid boundary vertices to minimise the likelihood of misassignment within and between participants (since parcellation and normalisation are never perfect).

We thank the reviewer for the graph theory remarks. First, the body part nodes in the current experiment are independent as they correspond to independent movement cues. That there is a relation between body part representations does not mean the nodes corresponding to the body part representations are not determined independently from each other. As a matter of fact, such between node relationship is exactly what is quantified with graph theory. However, the nodes are indeed not defined by an equal number of vertices. This is unfortunately the way different body parts are cortically represented. We now mention this issue in the discussion at lines L408-410.

- Can the authors report the number of vertices used for each body part, and show their location on a map?

Rather than including the number of vertices, which does not take surface curvature into account, we have added a figure with the surface area per body part representation per cortical area to the manuscript (Figure 2, see also reviewer #1). The cortical areas in Figure 2 correspond directly with the cortical areas from Figure 3, which makes an additional map redundant.

- Finally, performing a graph analysis on coefficients of linear correlations between average distances obtained for each body part does not reflect functional connectivity, which is usually what graph theory is used for in neuroscience. Rather, this analysis seems to assess a sort of simplified and inaccurate measure of 'overlap' between representations. And tools such as representational similarity analysis can inform us about how body part representations relate to each other. So, I struggle to see what this graph analysis brings. If I am missing something, please explain.

We thank the reviewer for this opportunity to elaborate on our decision to use graph theory. The reviewer argues that our graph theory analysis does not reflect functional connectivity. However, we would argue the opposite: the graph theory analysis does reflect functional connectivity; not between brain regions (as is often the case for graph theory in neuroscience), but functional connectivity between body part representations. We agree with the reviewer this specific use of graph theory resembles representational similarity, but that does not mean that representational similarity is the exclusive method for assessing the relationship between representations. Graph theory has a solid foundation in scientific analyses and here we argue that metrics such as 'degree', 'clustering coefficient', and 'betweenness centrality', can be used to represent 'uniqueness', 'cliqueness', 'influence', respectively of body part representations. Additionally, Louvain modularity is able to group specific body part representations together, resulting in highly intuitive modules for primary sensorimotor cortices, and deviating modules for cortical areas that are further up the functional hierarchy. We do not understand why, and therefore do not agree that, these metrics of functional connectivity are simplified and inaccurate. Thus, where representational similarity offers neuroscience an excellent method to assess the relationship between cortical representations, here we demonstrate how the validated method of graph theory, including its abundant repertoire of metrics, can be wielded for similar and transcending purposes Differences and similarities with respect to representational similarity analysis have now added to the discussion L484-489.

<u>Minor comments</u>:

- Figure 6 is too small

The figure has been improved upon (see reviewer #1), however its final size on print (rather than in a pdf-file) is currently not easily assessed by us.

- the authors should report the standard deviation for the age of the participants, as well as their handedness.

This information has been added.

- the authors should report head motion values (range, average, threshold used to exclude outliers etc)

For simplicity, we only mention the movement condition(s) with significantly larger head motion (i.e., the knee condition, see also reviewer #1). No outliers were excluded, as is mentioned in the manuscript (L723-729).

- For the point made lines 348-350 in the discussion, consider Ejaz et al, 2015.

We thank the reviewer for bringing this study to our attention and is now referenced with respect to this discussion (L373-374).

<u>Reviewer #3:</u>

Summary

This is a nicely presented manuscript summarising data from an 7T fMRI experiment and a novel approach to analysing the results. The aim of the study / approach is to shed light on the sensori-motor representations in different areas of human cortex. The method is a modification of the popular population receptive field method, motivated by a graph-theory approach. It manages to capture the fact that maps of sensory / motor response fields (if and when they exist) are don't have a known metric, but are likely to follow a consistent ordering. The non-rigid method presented here allows to estimate the weighted contribution from motion in different body region, regardless of the uneven (and unknown) mapping in sensory space

I have a couple of smaller comments and two questions which might require a little bit of additional thought, but I think overall, this is a very interesting paper that might be of interest to a wider audience.

General comment:

The way the model is presented appeals and I was intrigued by the graph theory analysis that was used to interpret and summarise the pRF fitting findings. On the data fitting / optimisation itself, I think the authors need to explain in a bit more detail how the allowing the different body area / cortical map territories to contribute in this non-rigid model fit is different from simply allowing a separate scale factor for each... thinking through this part of the analysis in detail: different body part responses can contribute to the predicted response in a weighted, rank-ordered way - if my understanding of this is correcrt, then the fitting should work equally well with a profile of y=1-x, x=[0..1], just the estimated d_i parameters would be different, modulo standard gaussian quantiles.... I am not saying this because I think the authors should do it the other way, but suggesting that maybe an additional diagram of a sample model profile showing the non-rigid placement of an example fit solution would help readers follow this logic a bit better. Additionally, knowing how often the d_i parameters bunch up / collapse on a small region of the parameter range would be important to know, as these numbers will be obscured by relying on the gaussian profile and only showing a summary version of the non-rigid part of the fit parameters.

We like to thank the reviewer for this very constructive comment. The reviewer is completely right in his/her description of our non-rigid pRF method, including the rank-ordered alternative. We have added an additional figure to the methods, Figure 11, which hopefully

clarifies the fitting procedure and derived pRF metrics. Per request, Figure 5 below shows the histogram of dxi-values.

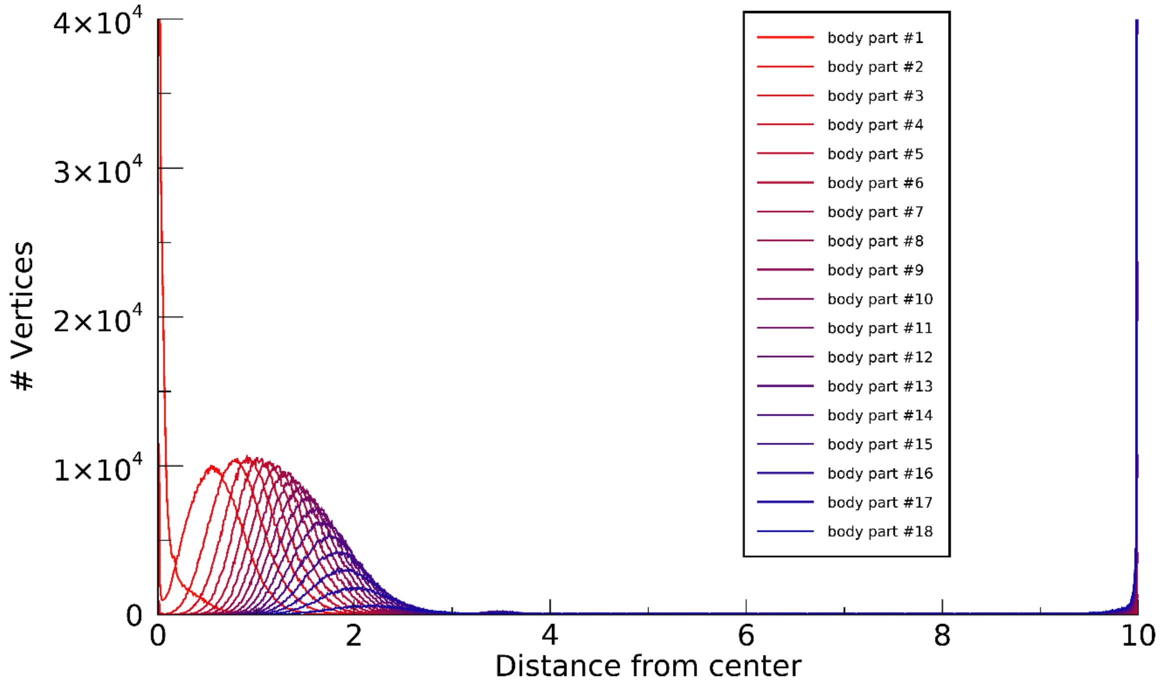

Figure 5. Histogram dxi-values. The separate lines indicate the frequency of dxi-values (i.e. distance from center) in a ranked order from the most central body part, i.e., body part #1, up to the most peripheral body part, i.e., body part #18, derived from all participants. Note that any body part could be the most central (or most peripheral) and that, thus, the different lines do not correspond to specific body parts, but to their ranked position within the response field. The line for body part #1 is equal to the bar plot presented in Figure 3 (apart from the axes ranges). Body parts with dxi-values around '10', reflect body part movements that did not result in an observable BOLD-response, or resulted in a negative BOLD response.

The regions of interest for the network analysis seem a bit ad-hoc (as the authors acknowledge). I understand the motivation for carving up the cortex into these sub-regions that appear to have a somato/mototopic map... but given my comments on appearance of map-like structure (from color maps) below and also the fact that other parcellations are readily available: should going with other parcellations (say, by standard anatomical criteria) not produce similar graph summaries? Or are the results presented here, dependent on / tuned to the choice of ROIs? Maybe explaining this in a bit more detail would help (it's on of the few places where I felt I had to go between results and methods section back and forth a couple of times to understand... so worth smoothing over this).

We like to thank the reviewer for this opportunity to elaborate on the choice of ROIs. With respect to readily available atlases, the current parcellation is actually based on the Brodmann parcellation for M1 and S1, while it is loosely based on Destrieux et al., *Automatic parcellation of human cortical gyri and sulci using standard anatomical nomenclature*, 2010 for the other areas. The reason behind combining multiple atlases in our study is that -to our knowledge- there exists no atlas with specific sensorimotor parcellations. Hence, other available atlases provide less relevant cortical parcellations, and often either very large and chunky, or overly precise and tiny cortical area parcellations. Therefore, we selected the ROIs as presented in the study on the basis of sensorimotor relevance. Does our choice in ROI affect the outcome? Yes, it probably does. We selected regions that are thought or known to be relevant in sensorimotor functioning. Therefore, we are able find somatotopic maps. Selecting ROIs on a different ground than sensorimotor relevance will decrease motor

pRF fits and subsequent metrics. We made sure to add clarifications in the introduction (L119), results (L148-151).

Specific comments:

I like the approach of showing the full maps of the estimated pRF centers and sized (unthresholded by a correlation or r^2 value). See Figures 2, 3... in particular, this allows everyone to see what the coverage of the fMRI slab was. This therefore avoids the common problem of "no response/value" hiding the fact that no data was acquired for those locations, rather than not passing some statistical criterion. However the flipside of this is that areas of cortex that are unlikely to be tuned to any aspects of sensorimotor function get assigned a value (for centers, ostensibly related to ~hand function... for pRF sizes, some middlish value). For me, two questions spring from this

1. there is clearly some regression / blurring of those estimates towards the mean of the parameter range. Is this an artefact of the optimisation (starting values?) or something else. One way to shed light on this would be to show the goodness of fit / r^2 values for everywhere... which presumably highlights S1/M1, S2, SMA and other areas -- I suggest the authors add at least a panel indicating a statistic like this in the main MS (+ more detailed info in the Supplementary Materials)

We have added descriptive statistics in the form of R^2-values per cortical area to the results (L149-153, see also reviewer #2), as well as a Supplementary Figure 3 to the manuscript showing F-statistic goodness-of-fit values plotted on the cortical surface meshes.

2. In both cases, the choice of colormap could probably be different. I am saying this as _some_ maps like jet() or hsv() / or similar maps in matplotlib are not perceptually equivalent at different points on the scale ... the effect is to create perceptual groupings in the displayed maps that don't reflect either the (quasi)-linear scale imposed by the categories (individual sites... or even face... to lower limbs). I think a more natural choice would have been something that has perceptually matched step for each categorical jump - or to go for a colormap that is made for increasing / decreasing scales. An additional detail here is that for many people, a mapping for hot colors to low values and blueish colors to high values (as in Figure 3) is probably the opposite of what they expect. For the size map 1...14, an increasing color map without categorical jumps seems definitely better.

We agree with the reviewer that the use of this specific color table for pRF size (Figure 4) is not optimal. Hence, this has been adjusted. However, we think that the pRF centers, as well as the somatotopic information in the graph theory figures is well represented by the currently used categorical color table: lower limb: red, upper limb: yellow, hand: green, and face: blue/purple. We acknowledge there are problems with the 'rainbow colortable' and that it is not well-suited for some types of color blindness. However, we think that for most readers this is an intuitive manner to depict the somatotopy, also because it has been used to depict somatotopic arrangements in previous scientific articles: e.g., Puckett et al;., 2020, Martuzzi et al., 2014, Sanchez-Panchuelo et al., 2018, Zeharia et al., 2015).
