# Supplementary material for: Moving in on human motor cortex. Characterizing the relationship between body parts with non-rigid population response fields
Source: PLoS Comput Biol. 2022 Apr 4;18(4):e1009955. doi: 10.1371/journal.pcbi.1009955 (PMC9009778; doi:10.1371/journal.pcbi.1009955)
Supplement: Attachment — Submitted filename: Rebuttal_V2.0.pdf [file pcbi.1009955.s016.pdf]

Manuscript Schellekens et al.
for PLOS Computational Biology

Reviewer #1
Thank you to the authors for a thorough reply. My major concerns have been addressed.

I would suggest that the authors include the plots of connectivity vs. pRF size in the supplement. I appreciated their discussion in their reply, and believe that the finding would be of interest to some readers. The positive correlation suggests that the author's definition of pRF size, i.e. representational spread across body parts, can predict connectivity, i.e. how related one pRF center is to another.
We would like to thank the reviewer for his/her time. The connectivity versus pRF size plots are added as Supplementary figure 7 and referenced in the manuscript at L351-352

I think the current version of Figure 6 is acceptable. My suggestion was to actually mark each subject in a different color point in the plot (rather than all points being one color), but I see that it adds enough visual noise that it may not be useful.
Then we thank the reviewer for the constructive suggestions, and keep the current Figure 6 of the manuscript *as is*.

I have not seen any code. The authors should be able to release the code used to actually form the graphs, compute the various metrics, and run the statistical tests.
The main code for the analysis can be viewed at: https://github.com/wschelle/NRpRF/

Reviewer #2

I would like to thank the authors for their efforts to address the points I raised. Even though I really value the authors' approach and their will to look at the relationship between body parts, something that has not been investigated enough by the community, the authors' answers are not convincingly addressing the issues I raised. I am thus not sure how much reliable are the results, rendering their interpretation difficult.

My point regarding the pseudo-randomisation of conditions has not been addressed in a satisfying way. I appreciate the authors' effort to show that the tSNR is not significantly different between runs, but it does not change the fact that splitting conditions into two separate runs introduces a bias in the inter-correlations between conditions (e.g., artificially increasing the coupling between hand and trunk conditions and artificially decreasing the coupling between hand and leg or hand and face conditions). Especially since no cross-validation is done here.
We agree with the reviewer that the task design may possibly have introduced a bias of body part relationship. Body parts that were cued for movement in the same run could have been unintentionally coupled to one another. This could be harmful for the estimation of body part distances per surface mesh vertex and, therefore, the pRF size estimation. Thus, the question arises whether body part distances on average differ for body parts presented within the same run, compared to the estimated body part distances presented during the other run. Therefore, we tested if such bias exists for our body part distance estimation.

Our original non-rigid analysis calculates 18 distances to a theoretical response field center (i.e. one for each body part) per surface vertex. The pRF center is based on the shortest distance. For this test we averaged the distances per pRF center (all vertices within the FOV), and then split the 18 distances in 2 groups: the first group represents 9 body part distances that were presented in the same run as the estimated pRF center, and the second group represents 9 distances of body parts presented during the other run relative to the pRF center body part. Using a Welch t-test we tested if the distances of body parts cued in the same run as the pRF center differ significantly from the distances of body parts cued in

the other run. We found no evidence for a bias in distance estimation between body parts presented within the same versus the different run as the pRF center: Welch $t_{(13)} = 0.342$, $p = 0.737$. Apart from the knee (which suffered from relatively increased head motion), body part distances appear homogeneously distributed across the 2 different runs (Figure 1). The test has been added to manuscript L204-208 & L698-702.

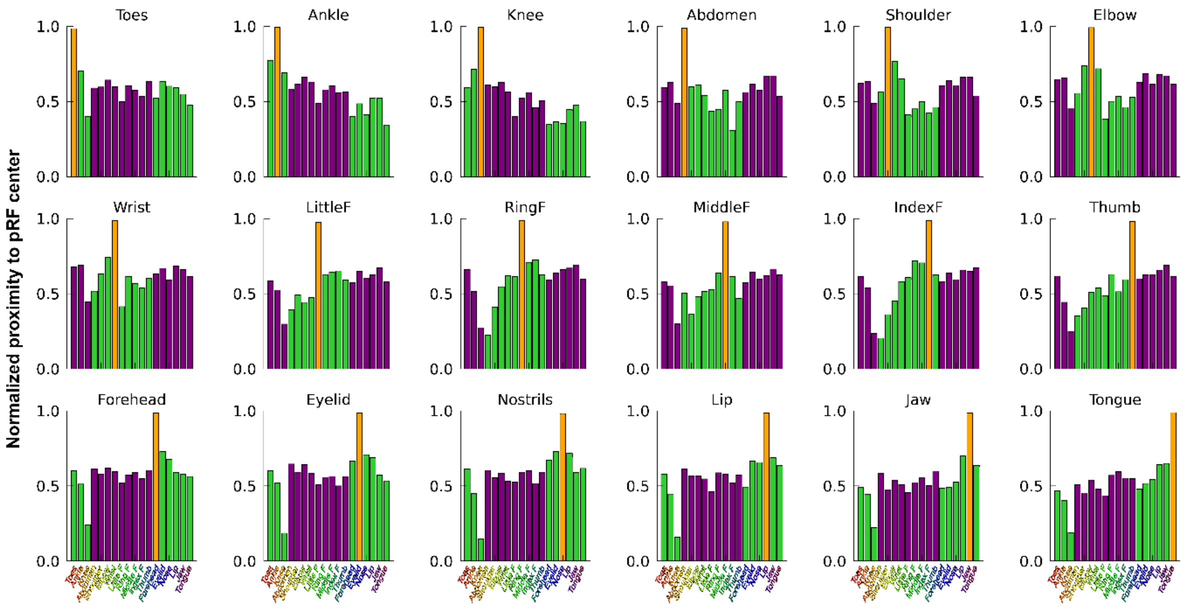

Figure 1: Task bias.
The normalized distances (1=center) for all included surface vertices are shown, averaged over the estimated pRF center for each body part. The estimated pRF center is presented by the orange bar and by default is closest to the pRF center (i.e. highest normalized proximity value). The body parts that were presented during the same run as the estimated pRF center are represented by the green bars, while the body parts presented in the different run as the pRF center body part are shown by the purple bars. There is no consistent preference (or averseness) for the green bars, which would indicate a task bias.

Together with the very variable number of vertices/surface area used for each node to compute the graph theory measures, that introduces another level of bias, I don't know how much the data can be trusted/interpreted. In addition, stating that only the knee condition resulted in significantly more head motion is not sufficient. Head motion could bias drastically the results too.
For our answer to the surface area used for graph theory measures, see the more detailed question posed by the reviewer below. Regarding head motion artefacts: we fully acknowledge that motion artefacts can be problematic for fMRI studies. However, this is true for all fMRI studies. Additionally, we acknowledge that our original answer with respect to the head motion topic was incomplete. We calculated the total displacement following any motor cue. The mean displacement across all subjects and motor conditions was 0.68 mm s.d. = 0.53 mm. The average displacement is significantly smaller than the acquired voxel size of 1.75 mm ($t_{(17)} = -8.53$ $p < 0.001$). Hence, there is no reason to suspect the presence of motion artefacts beyond the range of normal behavior of healthy volunteers. We, therefore, do not see any reason to cast additional doubt on our results. Possible motion artefacts in relation to the knee condition were already discussed in the article at lines L341-343.

I tried to suggest analyses that could be done to validate the dataset by replicating previous findings (by the authors and others), but I am open to any other suggestion if the authors find other ways of achieving this goal. The only thing replicated here physiologically speaking is the somatotopy in S1 and M1. And even at this level, no clear somatotopy is observed for the face parts in S1, when this would be expected, even with an active paradigm (see Root et al, 2021).

We disagree with the statement that no clear somatotopy was observed for the face parts in M1 and S1. Figure 5 of the manuscript clearly shows an average gradual shift of body parts, including face representations, across cortical locations. If the reviewer is referring to the apparent radial/polar cortical organization of facial feature representations visible in M1/S1 (Figure 3), then we would like to mention that a similar organization has previously been described in macaque S1 with electrophysiology recordings (Dreyer et al., 1975). We have added this topic to the discussion at L292-294

The smaller receptive fields in S1 compared to M1 (also suggested by the conventional pRF method) are not replicated, the relative size of RFs between body parts is not replicated (see more details below), nor is the cortical magnification of the different body parts. When it comes to active paradigms, other findings could be attempted to be replicated, such as the hand-mouth coupling that was reported in the parietal areas (see Stepniewska et al, 2009).

The trend of smaller pRF sizes for S1 compared to M1 is visible for both the conventional as well as the non-rigid approach. However, also for the conventional pRF method the difference in pRF size between M1 and S1 is not significant: Welch $t_{(13)} = 1.107$, $p = 0.288$.

With respect to the cortical magnification factor, it may be the case that our current results deviate from previous findings. However, it needs to be mentioned that many previous studies did not distinguish between the 5 fingers, and sometimes group the entire forelimb/upper limb. In order to assess whether our results deviate from previous findings, those body part specific metrics would need to be available.

We did not design an experiment to test specifically for hand-mouth coupling. However, from the graph theory figures, it can be observed that substantial functional connections exist between shoulder/elbow/wrist and the face/mouth in superior parietal cortex. Additionally, we putatively show a related finding: that the shoulder and elbow have an influential role in superior parietal cortex.

Could the authors show the individual's pRF center and size maps to see at least how consistent the results are?

Yes individual flat maps are added to this rebuttal and as Supplementary figures to the main manuscript (given that the editor allows it).

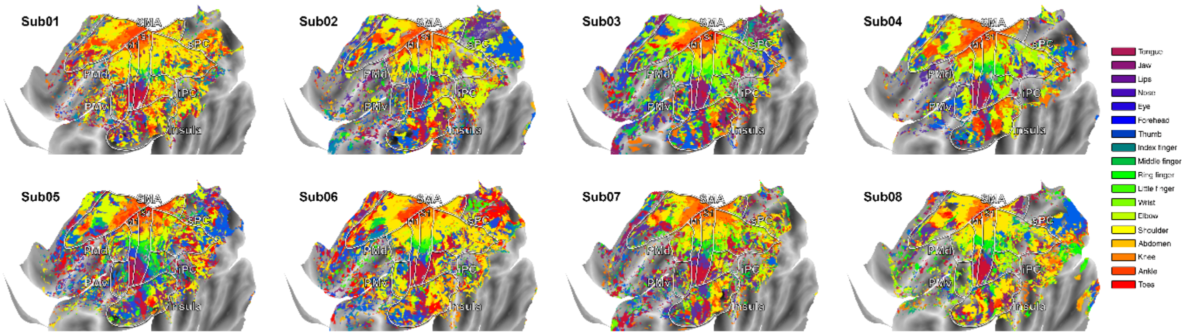

Figure 2. Individual pRF center maps. For each subject the pRF centers projected on a flattened cortex reconstruction are shown with different colors representing different body parts. The ROIs are denoted by the white lines and text on top of the maps.

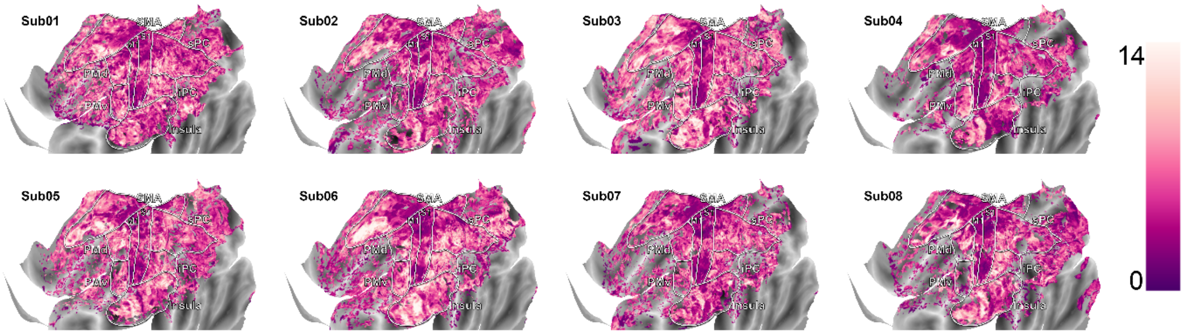

Figure 3. Individual pRF size maps. For each subject the pRF sizes projected on a flattened cortex reconstruction are shown. Darker hues represent smaller pRF sizes and lighter hues represent larger pRF sizes. The ROIs are denoted by the white lines and text on top of the maps.

I answer into more details to some points below:

- The design bias (i.e., separate runs) is difficult to account for posthoc indeed. Thus, my suggestion to find a control ROI, but I agree it's suboptimal.
We cannot alter the experimental design posthoc. However, we demonstrate here in this rebuttal that the separate runs did not artificially, and unintendedly, created a coupling between body parts of the same run (addressed in the first topic).

Regarding the authors' explications and choice in terms of visual instructions, I do not understand why pictures were used rather than simple written words displayed at the center of the screen. In addition to the eye movement issue, looking at body images could also affect and bias the coupling between the co-displayed body parts in parietal regions where multisensory integration is known to take place.
Our decision for illustrated motor cues was made to accomplish intuitive and quick motor responses. We feared that verbal instructions might have increased the motor onset latency and, additionally, could have introduced variability between body part onset latencies depending on word length. More task errors can, additionally, be expected for (mild) cases of dyslexia. However, if an unintended coupling caused by visual cues existed, it would be represented as a bias for body parts presented within the same run. As mentioned, we found no evidence for an experimental task design bias in our results.

The sentence corrected by the authors line 202-204 is still misleading. The authors should make it clear that despite the randomization, the fact the different body parts are tested in different runs could introduce artificial coupling and decoupling of body parts.
We have adjusted the sentence to:
"The movement order of body parts was pseudo-randomized to prevent an artificial coupling of body parts on the basis of the experimental design, despite that such artificial coupling may have persisted between separate runs. However, we found no evidence for an artificial coupling of body parts that were cued within a single run (Welch $t_{(13)} = 0.34$ $p = 0.737$)"

- To account for the bias introduced by the different surface area used for each node in the graph theory, the authors should limit their analysis to the same amount of vertices/surface area for all nodes/body parts (at the individual level) to be able to compare graph theory measures. It's not because body parts have different cortical magnification that the entire representation has to be taken into account to assess the graph theory measures.
We understand that using a different number of surface vertices to construct graph theory nodes is suboptimal. However, the reviewer's solution unfortunately runs into problems as well. If we were to select the same number of surface vertices for each body part per ROI,

then a substantial set of vertices for all but one body part will have to be discarded (i.e., to match the number of vertices of the body part with the smallest representation). How would we make such a selection? There is a high probability that selecting a subset of vertices out of all vertices with a specific body part preference will bias the outcome. Not only will the outcome likely be biased, it would not be an accurate display of body part connectivity, as large parts of cortex are discarded. We could try to salvage this problem by iterating the random selection of a minimum set of vertices many thousands of times, after which the results are averaged over the iterations. That, however, should theoretically converge towards the average of all vertices, which is similar to averaging over all vertices straight away as is performed in the current analysis. Finally, by iteratively discarding many vertices, a different graph may be constructed at every iteration. Not only is this problematic for visualization, it could introduce 'node islands' within the graph and 'Not a Number' (NaN) for the graph theory metrics. As a side note, this particular issue exists for all neuroscientific applications of graph theory, and would also exist for "Representational Similarity Analysis". The fact that not every ROI or feature is represented by the same number of data points, is simply innate to the human brain. Discarding powerful tools to investigate functional connectivity between ROIs or feature representations in the brain appears wasteful.

- Regarding the novel pRF method, while I really value the will to not have any a priori regarding the relationship between body parts, it is important to note and discuss that this method assumes a fixed pRF size in their model (not to be confused with the pRF size estimated based on the distances), which is not much valid physiologically speaking, between body parts but also when looking at more integrative parts of the cortex (as suggested by the large under-estimation of the pRF size compared to the conventional pRF method, as quantified in Figure 4 in the answers to reviewers, figure that I think should be added as Supplemental figure and discrepancy that should be further discussed than actually done).

We think that the fixed width of the Gaussian function is adequately described and discussed both in the introduction (L87-91), as well as in the discussion (L415-426). We clearly state that the non-rigid pRF method makes this assumption, and that alternative assumptions might yield different results.

Furthermore, we disagree that the non-rigid pRF methods under-estimates pRF sizes. Rather, it is the other way around: the conventional pRF method over-estimates pRF sizes. The conventional pRF methods assumes a rigid order of body parts. If a neuronal population represents body parts that are not directly adjacent to each other on this rigid body order space (e.g. the hand-mouth coupling mentioned earlier by the reviewer), then the conventional pRF method is forced to encompass the body parts in between. Therefore, the conventional pRF method artificially widens the pRF size and can be forced to include body parts that did not show signs of activity within the response field. We added further clarifications to the discussion at L436-439.

Even though I agree we are drastically lacking human data when it comes to multiple body parts, RFs size has been investigated using electrophysiology in monkeys and other animal model. And RFs in S1 are massively larger for example for the trunk or face areas than at the finger level (see e.g., Conti et al, 1986; Dreyer et al, 1975). This difference in RFs size is hypothesised to account for the massive differences in discrimination between body parts, with very poor discrimination at the trunk and leg level, and very high discrimination at the fingers, face or foot sole's glabrous skin (see Mancini et al, 2014, Strzalkowski et al, 2018). Even though I agree that an active paradigm is less likely to tap into detailed aspects of S1 structure, the activity observed in S1 when moving a finger is very similar to what is observed during a passive paradigm (see Berlot et al, 2019; Sanders et al, 2019). And this activity is still reflecting the contribution of both the efferent copy (i.e., prediction of sensory inputs expected by the movement) and the actual sensory input that would both be still more refined for the fingers than for the trunk or legs. Meaning that even if indeed RF sizes

are expected to be larger than with a passive paradigm, the relative differences between body parts, based on what we know from both electrophysiological and psychophysical studies, should be preserved, or at least not reversed (as suggested by the present results).

We thank the reviewer for acknowledging the importance of human data on the topic of motor control and body part relationships. The way humans use their body parts, and hand/fingers in particular, may have led to unique cortical activation patterns in relation to body part motor control. That said, our current study does not intent to seek contradictions with existing literature, but we do understand that the relatively enlarged pRF size for the fingers seems to be in direct contradiction with existing (animal) models on receptive field size. However, there are certain important topics to be considered with respect to sensorimotor pRF sizes.
We mainly would like to argue that our results may not deviate from previous findings and existing understandings as much as it might appear on first sight: First, many previous (animal) somatosensory studies lack a numeric quantification of receptive field size (including the studies mentioned by the reviewer). This makes it difficult to conclude whether our pRF size estimates deviate substantially from earlier findings. Second, receptive/response field estimates are dependent on the stimulated (active or passive) body parts. For example, the study by Conti et al., 1986 report 'large' bilateral receptive fields for trunk representations in S1. Our study does not explicitly contradict this earlier finding. What is important here, is that we included only one midsection body part (i.e. abdomen). The neuronal populations we found to prefer the abdomen might also have large bilateral response/receptive fields, including a large area of the skin covering the trunk and movements performed by e.g. the spine. Our pRF size simply was not estimated with these specific properties, mainly because active paradigms in an MR-environment are restricted in the type and extent of movements. That does not mean, that our pRF estimate is incorrect. We merely demonstrate that, given our 18-body part motor task, this is how the cued body parts are represented in response fields across multiple motor-related cortices in humans. Hence, our pRF size estimates for the fingers should be interpreted as such (i.e., with respect to the 18 cued body parts) with proportionally many upper limb body parts that can be incorporated in the response fields. Note, that in units of mm^2 area of the skin, neuronal populations of the fingers may actually have relatively small receptive field sizes, since the fingers are relatively small. It, however, makes no sense to describe motor response fields in units of mm^2 skin area, as the skin itself is not cued for movement. Instead we argue that motor pRFs should be described in terms of moveable body parts, or joints, which could sometimes lead to unintuitive pRF size estimates.
In conclusion, our results may differ from previous sensorimotor organizational descriptions, but that may be expected on the basis of different species investigated and the respective differences in motor behavior. However, overall we argue that our results are not in conflict with existing theories. We primarily present motor pRFs as a function of moveable body parts, rather than as a function of a passively excitable area of the skin. We have elaborated on this topic in the discussion L326-329.

- the authors should discuss the low percentage of variance explained by their model.
The relatively low variance explained is mainly caused by two factors: 1. Slightly variable BOLD hemodynamic response function (HRF) curve onsets relative to the motor cue onset. 2. We observed deviating HRFs with respect to the canonical HRF used in the model. Particularly, the observed motor HRFs displayed a substantially larger undershoot, than was accounted for by the model. This is now mentioned in the discussion at L441-445

- thank you for letting me know that only 1% of vertices are affected by the average procedure. Very good news. Adding this in the methods would reassure the reader.
It has been added to the procedures L571.

Reviewer #3
The authors have made great and very thorough efforts to address my comments (and I saw they took equal care with for the comments raised by the other two reviewers). The revised manuscripts addresses the points I have raised - I have no further concerns.

We would like to thank the reviewer for his/her time and the constructive suggestions!
