# Supplementary material for: Moving in on human motor cortex. Characterizing the relationship between body parts with non-rigid population response fields
Source: PLoS Comput Biol. 2022 Apr 4;18(4):e1009955. doi: 10.1371/journal.pcbi.1009955 (PMC9009778; doi:10.1371/journal.pcbi.1009955)
Supplement: Attachment — Submitted filename: Review manuscript Schellekens et al.docx [file pcbi.1009955.s013.docx]

**Review manuscript Schellekens et al.**

**for PLOS Computational Biology**

In the manuscript “**Moving in on human motor cortex. Characterizing the relationship between body parts with non-rigid population Response Fields**”, Schellekens and colleagues investigate the relationship between body part representations in sensorimotor cortices, using a non-rigid population Response Field (pRF) method that they developed, combined with graph theory. From the pRF mapping, the authors report somatotopic organisation in all included motor areas, and a higher selectivity in primary cortices than in secondary ones. Applying graph theory on this data, they report unique response profiles for the knee, that digits of the hand cluster around the ring finger and a central role for the shoulder and elbow. The authors conclude that these results reveal associations among body parts at the cortical level that are in agreement with intuitive notions of body part usage. Finally, the authors emphasise that combined with graph theory, their new pRF method allows to investigate neuronal population response fields, when the relationship among selected functions is not apriori known.

The study is well-designed, well-written and using cutting-edge techniques to investigate a very interesting question that has not been addressed to my knowledge. However, some methodological aspects make it difficult to understand what can be gained from all these impressive analyses, apart from the new pRF method they describe and validate using the conventional pRF method.

Here are my major comments:

**Related to the design:**

- lines 506-507: ‘The functional session was split in 2 runs (torso/arm/hand and head/leg, figure 1)’.

It seems to me the authors cannot directly compare in their analyses the data from the leg/face and the hand/trunk since it was acquired in different runs. The authors should have presented all conditions in each run, to avoid introducing a bias both in the pseudo-randomisation of the conditions (not fully pseudo-randomised here), and in the run-dependent variability/noise introduced in the design. Rescaling to percent BOLD signal change does not fully account for that.

- lines 205-207: ‘We ensured that the movement order of body parts during scanning was randomized to prevent body parts from being coupled to one another on the basis of the experimental design.’

I understood from the lines 506-507 cited above that the torso/arm/hand conditions were tested in one run and the head/leg conditions in another. If I misunderstood, please rephrase lines 506-507 because it is misleading this way. If I understood correctly, please rephrase lines 205-207 to make explicit that all conditions were not fully counter-balanced and that as a result some body parts might be more coupled than other (e.g., leg/head might be artificially more coupled than leg/hand).

**Related to the new pRF method:**

- If the conventional approach gives similar maps and pRF sizes, could the authors explain what is the point of this non-linear approach, at least for the sensorimotor regions?

- The authors should take a control ROI that is not sensorimotor to make sure the method is not introducing any biases.

- The authors have not tested all possible body parts, so by selecting the lowest distance to the center, the authors may have misassigned pRF centers. Indeed, if the lowest distance exceeds a certain distance, the vertex should not be assigned a pRF center. The authors could for instance use the goodness of fit to select which vertices to keep for the pRF mapping and the related measures.

- The mean and range of goodness of fit obtained across all vertices and ROIs should be reported.

- Can the authors explain better what they mean line 579-581: ‘In case of multiple body parts with the lowest distance to x0, the pRF center was calculated as the mean index of the multiple body parts and rounded to the nearest whole integer’. I understand that if for instance body parts 1, 6 and 8 (e.g., toes, elbow, little finger) ended up having the same lowest distance to x0, the authors took the mean of their indexes (here 5), and assigned that vertex to body part 5, which is the shoulder. If I understood correctly, this is inserting a lot of biases and assuming only neighbouring body parts would have such conflicting issues. It may artificially make the distances and pRF maps more linear/smooth.

- The fact that the authors report large pRF sizes for the fingers also in S1 is counter-intuitive, both in terms of receptive size and in terms of selectivity. Finger representations in S1 are known to be quite selective and to display the smallest receptive fields.

To understand a bit better and compare to pre-existing results, it would be interesting to report the inter-finger data. Do the authors see something similar to what was reported by Puckett et al, 2020? Do they observe different pRF size across fingers? Do they observe different pRF size within SI (along the antero-posterior axis)? It would be interesting to include BA2 in the S1 ROI to compare the different subregions (i.e., BA3b,1,2). In this regard, the conventional approach seems to give more consistent pRF sizes, with the smallest pRF sizes located in S1 and it seems more in BA3b,1 than in BA2. The authors should discuss the results observed in S1.

- lines 594-597: ‘Since body parts were only fitted in one side of the Gaussian shaped response field, only body parts that were in range of half the width at half maximum were included in the pRF size calculation’.

It is not clear which body parts we included/kept for the pRF size measure. The fact that some body parts were not taken into account should be reported in the main text, as well as which body parts were in the end used for that measure in each ROI.

- The mention ‘although pRF sizes estimated by the conventional pRF method tend to be larger on average’ is not informative to me and even misleading since the conventional approach seems to result in smaller pRF sizes for S1, M1 and the insula, and larger sizes for the other ROIs.

**Related to the graph theory analysis:**

- Graph theory requires nodes to be independent from each other (i.e., to correspond to independent structures or representations) and of the same size (i.e., same number of voxels) to avoid biasing the results towards one node or another. The way nodes are defined is highly affecting the outcome measures, both quantitatively and qualitatively (see e.g., Zalesky et al, 2010; Fornito, Zalesky & Bullmore, 2010; Hayasaka & Lauriento, 2010; Wang et al, 2009). Here the authors selected the vertices showing significant goodness of fit for each body parts (lines 611-613), but this is likely to results in different number of vertices. And care should be taken to avoid boundary vertices to minimise the likelihood of misassignment within and between participants (since parcellation and normalisation are never perfect).

- Can the authors report the number of vertices used for each body part, and show their location on a map?

- Finally, performing a graph analysis on coefficients of linear correlations between average distances obtained for each body part does not reflect functional connectivity, which is usually what graph theory is used for in neuroscience. Rather, this analysis seems to assess a sort of simplified and inaccurate measure of ‘overlap’ between representations. And tools such as representational similarity analysis can inform us about how body part representations relate to each other. So, I struggle to see what this graph analysis brings. If I am missing something, please explain.

Minor comments:

- Figure 6 is too small

- the authors should report the standard deviation for the age of the participants, as well as their handedness.

- the authors should report head motion values (range, average, threshold used to exclude outliers etc)

- For the point made lines 348-350 in the discussion, consider Ejaz et al, 2015.
