# Supplementary material for: Moving in on human motor cortex. Characterizing the relationship between body parts with non-rigid population response fields
Source: PLoS Comput Biol. 2022 Apr 4;18(4):e1009955. doi: 10.1371/journal.pcbi.1009955 (PMC9009778; doi:10.1371/journal.pcbi.1009955)
Supplement: Attachment — Submitted filename: Review2 manuscript Schellekens et al.docx [file pcbi.1009955.s015.docx]

**Review2 manuscript Schellekens et al.**

**for PLOS Computational Biology**

­

I would like to thank the authors for their efforts to address the points I raised. Even though I really value the authors’ approach and their will to look at the relationship between body parts, something that has not been investigated enough by the community, the authors’ answers are not convincingly addressing the issues I raised. I am thus not sure how much reliable are the results, rendering their interpretation difficult.

My point regarding the pseudo-randomisation of conditions has not been addressed in a satisfying way. I appreciate the authors’ effort to show that the tSNR is not significantly different between runs, but it does not change the fact that splitting conditions into two separate runs introduces a bias in the inter-correlations between conditions (e.g., artificially increasing the coupling between hand and trunk conditions and artificially decreasing the coupling between hand and leg or hand and face conditions). Especially since no cross-validation is done here. Together with the very variable number of vertices/surface area used for each node to compute the graph theory measures, that introduces another level of bias, I don’t know how much the data can be trusted/interpreted. In addition, stating that only the knee condition resulted in significantly more head motion is not sufficient. Head motion could bias drastically the results too.

I tried to suggest analyses that could be done to validate the dataset by replicating previous findings (by the authors and others), but I am open to any other suggestion if the authors find other ways of achieving this goal. The only thing replicated here physiologically speaking is the somatotopy in S1 and M1. And even at this level, no clear somatotopy is observed for the face parts in S1, when this would be expected, even with an active paradigm (see Root et al, 2021). The smaller receptive fields in S1 compared to M1 (also suggested by the conventional pRF method) are not replicated, the relative size of RFs between body parts is not replicated (see more details below), nor is the cortical magnification of the different body parts. When it comes to active paradigms, other findings could be attempted to be replicated, such as the hand-mouth coupling that was reported in the parietal areas (see Stepniewska et al, 2009).

Could the authors show the individual’s pRF center and size maps to see at least how consistent the results are?

I answer into more details to some points below:

- The design bias (i.e., separate runs) is difficult to account for posthoc indeed. Thus, my suggestion to find a control ROI, but I agree it’s suboptimal.

Regarding the authors’ explications and choice in terms of visual instructions, I do not understand why pictures were used rather than simple written words displayed at the center of the screen. In addition to the eye movement issue, looking at body images could also affect and bias the coupling between the co-displayed body parts in parietal regions where multisensory integration is known to take place.

The sentence corrected by the authors line 202-204 is still misleading. The authors should make it clear that despite the randomization, the fact the different body parts are tested in different runs could introduce artificial coupling and decoupling of body parts.

- To account for the bias introduced by the different surface area used for each node in the graph theory, the authors should limit their analysis to the same amount of vertices/surface area for all nodes/body parts (at the individual level) to be able to compare graph theory measures. It’s not because body parts have different cortical magnification that the entire representation has to be taken into account to assess the graph theory measures.

- Regarding the novel pRF method, while I really value the will to not have any a priori regarding the relationship between body parts, it is important to note and discuss that this method assumes a fixed pRF size in their model (not to be confused with the pRF size estimated based on the distances), which is not much valid physiologically speaking, between body parts but also when looking at more integrative parts of the cortex (as suggested by the large under-estimation of the pRF size compared to the conventional pRF method, as quantified in Figure 4 in the answers to reviewers, figure that I think should be added as Supplemental figure and discrepancy that should be further discussed than actually done). Even though I agree we are drastically lacking human data when it comes to multiple body parts, RFs size has been investigated using electrophysiology in monkeys and other animal model. And RFs in S1 are massively larger for example for the trunk or face areas than at the finger level (see e.g., Conti et al, 1986; Dreyer et al, 1975). This difference in RFs size is hypothesised to account for the massive differences in discrimination between body parts, with very poor discrimination at the trunk and leg level, and very high discrimination at the fingers, face or foot sole’s glabrous skin (see Mancini et al, 2014, Strzalkowski et al, 2018). Even though I agree that an active paradigm is less likely to tap into detailed aspects of S1 structure, the activity observed in S1 when moving a finger is very similar to what is observed during a passive paradigm (see Berlot et al, 2019; Sanders et al, 2019). And this activity is still reflecting the contribution of both the efferent copy (i.e., prediction of sensory inputs expected by the movement) and the actual sensory input that would both be still more refined for the fingers than for the trunk or legs. Meaning that even if indeed RF sizes are expected to be larger than with a passive paradigm, the relative differences between body parts, based on what we know from both electrophysiological and psychophysical studies, should be preserved, or at least not reversed (as suggested by the present results).

- the authors should discuss the low percentage of variance explained by their model.

- thank you for letting me know that only 1% of vertices are affected by the average procedure. Very good news. Adding this in the methods would reassure the reader.
