# Supplementary material for: Moving in on human motor cortex. Characterizing the relationship between body parts with non-rigid population response fields
Source: PLoS Comput Biol. 2022 Apr 4;18(4):e1009955. doi: 10.1371/journal.pcbi.1009955 (PMC9009778; doi:10.1371/journal.pcbi.1009955)
Supplement: Attachment — Submitted filename: RebuttalV3.0.docx [file pcbi.1009955.s017.docx]

*Reviewer #1: Thank you for uploading some version of the code. I am changing my recommendation to Accept since it now meets the journal requirements.

I would like to draw the authors' attention to these recommendations from the journal:

"Authors are encouraged to share their code in a way that follows best practice and facilitates reproducibility and reuse. Authors are encouraged to:
...Share clear documentation alongside the code that details any information needed to run the code, for example dependencies.
State how the code can be accessed in the Data Availability Statement of their manuscript."*

**Documentation: It seems that the .pro files use Qt. This is not very standard, so some example commands would be useful. In addition, I encourage the authors to document code dependencies, framework version, install requirements, etc. I can already see a couple functions which seem to be dependencies not included or listed anywhere in the repository (e.g. `mpfitfun`).**

We thank the reviewer for these helpful suggestions, and a link to the code is supplied in the data availability statement. We have expanded the documentation, although we are in the process of developing code that is easier to use for anyone.

**Access: Please include the repo link somewhere where it will be accessed with the manuscript (e.g. the Data Availability Statement.)**

The data availability statement is updated with data access information.

*Reviewer #2: I would like to thank the authors for their renewed efforts to address the points I raised.
I answer to some points below:*

**- Regarding the design bias issues, thank you for making these distance comparisons to evaluate the presence of potential biases. I think it’s a good way to make sure no massive bias was introduced indeed. However, this global measure (across the whole FOV) is unlikely to detect whether these design issues are impacting in more specific ways some cortical regions over others (depending on their function). This cannot be account for a posteriori and hinders interpretation in terms of sensorimotor organisation and relationship between body parts across cortical regions, except if the data is analysed and interpreted separately for the two sets of body parts (i.e., lowerlimb/face on one side and torso/upperlimb on the other).**

The presentation of body parts was split in two halves (hand/arm/torso and face/leg), which indeed could have caused a coupling of body parts by task design. This could hinder the interpretation of our results. Additionally, the reviewer correctly states that we cannot correct for a putative design bias a posteriori. However, we are not trying to correct for a task design bias. We merely tested the hypothesis that a task design bias was observable in the fMRI data, for which we found no significant evidence. We expanded on this topic in the discussion at lines L385-395

**- Regarding the discrepancies I highlighted relative to previous sensorimotor work, I appreciate the authors’ argument that an active paradigm may yield different estimates and acknowledged that some (e.g., pRF size) are indeed likely to be globally larger (since movements elicit more widespread tactile feedbacks). However, whether an active or passive paradigm is used, the general features of S1 and M1 (i.e., relative differences in cortical magnification, for recent papers see Roux et al, 2018/2020 with intracortical recording or Willoughby et al, 2021 with quantified magnification using fMRI) are preserved. The authors’ argument that “the skin itself is not cued for movement” highlights a misunderstanding of the sensorimotor system. The skin is continuously stimulated and stretched throughout any movement, providing enormous tactile feedbacks, which are crucial to adjust our actions (and increasingly attract interest for Brain-Machine interfaces and robotic devices for that reason). Tactile feedbacks that are processed by S1 in particular, whether inputs are triggered by passive touch or by movement. So, comparison/validation with previous knowledge about S1 organisation is legitimate.**

Indeed, tactile stimulation may contribute to our findings, but separation of motor and somatosensory function requires further research (which is challenging and will likely require invasive techniques). We do believe that the current results cannot be explained solely on the basis of tactile feedback and that the motor system does display multi-body-part integration. We added reference to somatosensory-motor literature in L337-346.

**- Regarding face somatotopy, several conflicting results were found in humans since Dreyer et al, 1975, but the most recent work using both imaging and intracortical recordings point towards an upward organisation of the face (Root et al 2021, Roux et al, 2018, 2020). Note that an inverse face organisation is found in monkeys (including macaques as in Dreyer 1975), with instead the lower face neighbouring the hand region (e.g., Manger et al, 2996). So, these cannot be used as a reference for humans (and thus to support present findings).**

On average, we too find an upward somatotopic organization of the face. However, on the basis of visual inspection of the group pRF center maps plotted on an inflated average brain surface (Figure 3), a radial organization can be appreciated similar as described by Dreyer et al., 1975. We merely wanted to present the possibility that the somatotopic face organization based on “average cortical coordinates” may be an oversimplification, and that a radial face somatotopy might be closer to the truth. Thus, with respect to this specific topic we do not seek support for our results among these previous studies, but rather wish to discuss different somatotopic organizational possibilities. We have made this clearer at L292-300.

**- Regarding the graph theory issue, I agree with the authors that the same issue applies to other analyses such as RSA, which is why when comparing different parts of the sensorimotor system within participants (i.e., like the comparison between different body part representations), care is taken to select the same amount of voxels/vertices. The point of such analyses is to go beyond simple map descriptions (where magnification, RF size, overlap etc can be described), to investigate the relationship between representations. To do so, potential confounds such as differences in magnification need to be accounted for by selecting the same amount of data, using specific criteria to define them in a similar way across conditions (i.e., x most selective ones or x voxels around the peak/CoG of the representation for instance). Otherwise, we end up mixing several characteristics and cannot compare/interpret the data.**

We decided to add an additional graph-theory analysis to the supplementary material, which equalizes the number of data points per graph node (i.e., equi-representational graph theory analysis). This way, the reader can appreciate and compare between the different representational graph theory approaches (see Supplementary material I).

To correct for differences in representational size for the equi-representational analysis, we performed the graph theory analysis iteratively on a random selection of cortical surface vertices. For each subject and each ROI we determined the minimum number of vertices that allowed for the body part graph theory analysis to be based on an equal number of data points. On average, approximately 81% (s.d. = 3%) of all significantly activated surface vertices were discarded to perform the equi-representational body part graph theory analysis. The graph theory metrics “connectivity”, “clustering coefficient”, and “betweenness centrality coefficient” were calculated as usual, as was the qualitative measure for Louvain modularity. This procedure was repeated 10.000 times, each time randomly selecting a matched subset of vertices for each body part representation per subject and ROI. All calculated graph theory metrics were averaged over the number of iterations, and were tested for statistical significance by means of the originally described statistical analysis.

Even though the equi-representational graph theory analysis leads to different graphs being created at every iteration, the pattern that emerges from the averaged iterations resembles the original graph theory approach (which is what we predicted in our previous correspondence). However, there were several deviations in terms of statistical significance, which are also listed here: Connectivity (degree) for the little finger is now also significantly smaller compared to other body parts (t_(119)_ = -4.83, p < 0.001). Area PMv does not display significantly larger connectivity values (t_(49)_ = 1.84, p = 0.072), whereas area iPC does show significantly larger connectivity values (t_(49)_ = 4.60, p < 0.001) The ankle displays a significantly larger clustering coefficient (t_(119)_ = 2.57, p = 0.011), while the ring finger clustering coefficient is not significant (t_(119)_ = 0.28, p = 0.783). In addition to the shoulder and the wrist, the tongue now also displays significantly larger betweenness centrality coefficients (t_(119)_ = 4.85, p < 0.001). However, PMv does not show significantly smaller betweenness centrality coefficients in the equi-representational graph theory analysis (t_(49)_ = -1.76, p = 0.085).

In conclusion, the equi-representational graph theory analysis produces comparable metrics compared to the original complete representational graph theory analysis. Several deviations in terms of statistical significance were found for some of the body parts and cortical areas, possibly indicating conditions on the border of statistical significance, and cortical areas with relatively heterogenous neuronal populations (e.g., PMv, also discussed at L421-423).

**- The editing lines 436-439 (“This finding likely illustrates that the conventional pRF model is forced to widen its Gaussian shape to encompass body parts that are not directly adjacent to each other with respect to the cortical homunculus ordering of body parts (e.g. a hand-mouth coupling in parietal cortex [89]) is misleading. It suggests that conventional pRF models might distort things, while it actually fits better what we know from electrophysiological studies (i.e., increased integration between body parts in higher order areas, as suggested by ref 89 and others, see Iwamura’s work).**

We thank the reviewer for these comments. Our non-rigid pRF model was developed to avoid somatotopic assumptions about internal RF organizations. Therefore, it is well-suited to investigate whether the conventional Gaussian RF model is adequate for sensorimotor activity. As stated at lines L460-462, the conventional pRF model works better for primary sensorimotor areas than for cortical areas that are higher up the functional hierarchy. Thus, we do not argue that the conventional pRF model presents a distorted sensorimotor representation by definition. However, the conventional pRF model may not be able to fully account for the complexity of internal RF structures of neuronal populations, as may occur in cortical areas beyond primary sensorimotor cortex. We have adjusted the aforementioned sentence to make it clearer that this issue mostly applies to higher-order cortical areas beyond primary sensorimotor cortex.
