# Supplementary material for: Moving in on human motor cortex. Characterizing the relationship between body parts with non-rigid population response fields
Source: PLoS Comput Biol. 2022 Apr 4;18(4):e1009955. doi: 10.1371/journal.pcbi.1009955 (PMC9009778; doi:10.1371/journal.pcbi.1009955)
Supplement: Attachment — Submitted filename: Rebuttal_V4.0.docx [file pcbi.1009955.s018.docx]

*Reviewer #2: I would like to thank the authors for the additions made to address the points I raised. In particular for doing the equi-graph analysis. I only have a few remaining requests for clarification and transparency:*

**- Thank you for adding the paragraph lines 385-395. Please specify that the bias you looked for was assessed across the whole FoV (line 388): e.g., “However, we specifically tested for an unintended task design bias, for which we found no evidence when taking into account the whole field of view”. Idem line 207, e.g., “However, we found no evidence for an artificial coupling of body parts that were cued within a single run across the whole field of view (Welch t(13) = 0.34 p = 0.737).”**

Done.

**Please add the bias estimation analysis and plot presented in the previous review as supplementary material and figure and refer to it in the main text. As explained in my previous comment, the fact that you do not find a consistent bias across the whole FOV does not preclude region-specific biases (that might cancel each other by averaging across the FOV).**

Done.

**- Please provide a quantification of head motion at the beginning of the results section when you say you found more motion for the knee condition line 153 (i.e., mean +/- sd motion for knee vs mean +/- sd motion for other conditions).**

Done.

**- Please mention at the beginning of the results section (either line 150, or in Table 1) that different body parts are tested in the two runs. Idem line 205-206. Make it explicit that different body parts are tested in the separate runs, thank you.**

Done.

**- line 297, please consider Moulton et al, 2009 to add to Dreyer’s reference (for more recent reference in humans).**

Done.
